# Effectiveness Modelling and Economic Evaluation of Primary HPV Screening for Cervical Cancer Prevention in New Zealand

**DOI:** 10.1371/journal.pone.0151619

**Published:** 2016-05-17

**Authors:** Jie-Bin Lew, Kate Simms, Megan Smith, Hazel Lewis, Harold Neal, Karen Canfell

**Affiliations:** 1 Cancer Research Division, Cancer Council New South Wales, New South Wales, Australia; 2 Public Health Physician, Wellington, New Zealand; 3 National Screening Unit, Ministry of Health, Wellington, New Zealand; 4 School of Public Health, Sydney Medical School, University of Sydney, Sydney, Australia; Istituto Nazionale Tumori, ITALY

## Abstract

**Background:**

New Zealand (NZ) is considering transitioning from 3-yearly cervical cytology screening in women 20–69 years (current practice) to primary HPV screening. We evaluated HPV-based screening in both HPV-unvaccinated women and cohorts offered HPV vaccination in New Zealand (vaccination coverage ~50%).

**Methods:**

A complex model of HPV transmission, vaccination, cervical screening, and invasive cervical cancer was extensively validated against national population-based datasets. Sixteen potential strategies for HPV screening were considered.

**Results:**

Most primary HPV strategies were more effective than current practice, for both unvaccinated women and cohorts offered vaccination. The optimal strategy for both groups was 5-yearly HPV screening in women aged 25–69 years with partial genotyping for HPV 16/18 and referral to colposcopy, and cytological triage of other oncogenic types. This is predicted to reduce cervical cancer incidence and mortality by a further 12–16% and to save 4–13% annually in program costs (excluding overheads). The findings are sensitive to assumptions about future adherence to initiating screening at 25 years.

**Conclusion:**

Primary HPV screening with partial genotyping would be more effective and less costly than the current cytology-based screening program, in both unvaccinated women and cohorts offered vaccination. These findings have been considered in a review of cervical screening in NZ.

## Introduction

The New Zealand (NZ) National Cervical Screening Programme (NCSP) was established in 1990 [1]. The programme recommends 3-yearly routine screening with liquid-based cytology (LBC) for 20–69 year-old women, with human papillomavirus (HPV) triage testing for low-grade (ASC-US/LSIL) cytology in women 30+ years [1], and a modified version of the Bethesda System is used for cytology classification. The NZ National HPV Immunisation Programme was introduced in September 2008, offering free HPV vaccine for females born in 1990 or later; school-based immunisation for 12–13 year old girls commenced in most regions in 2009 [2]. Three-dose coverage achieved by the program in cohorts born in 1991–2000 is approximately 48–56% nationwide [3].

Consideration of a transition to primary HPV testing for cervical screening in NZ has been prompted by the emergent international evidence on the increased effectiveness of primary HPV screening compared to cytology. Women who test negative for cervical HPV infection have a substantially lower risk of later developing high-grade cervical intraepithelial neoplasia (CIN 2/3 and CIN3+) [4,5,6,7] and cervical cancer [8] compared to women with a negative cytology result. Cost-effectiveness analyses in various settings have also found that screening with primary HPV testing is likely to be cost-effective, or even cost-saving, when compared to cytology-based screening [9,10,11]. The Netherlands, Australia and Italy are now implementing the transition to HPV screening. The Ministry of Health, Welfare and Sport of the Netherlands recently announced a review of the existing screening program and a plan to adopt primary HPV testing from January 2017 [12]. All cervical screening programs in Italy are scheduled to complete the transition to 5-yearly HPV screening in women aged 30/35 to 64 years based on the National Prevention Plan 2014–2018 [13]. Based on the findings of a systematic review and effectiveness modelling and economic evaluation, the National Cervical Screening Program in Australia will transition from May 2017 to 5-yearly primary HPV screening, using a test with partial genotyping for HPV 16/18 and reflex liquid-based cytology (LBC) triage for women with other oncogenic infection [14]. As a sentinel experience for programme change, Australia is also implementing a major trial of primary HPV screening in the partially HPV-vaccinated population, which is known as *Compass* (NCT02328872). A parallel pilot service evaluation project using primary HPV screening in NZ, known as *‘Compass NZ’* (ACTRN12614000714684) has also completed recruitment.

In this context, in 2015 New Zealand initiated a process of policy review of the NCSP to consider a potential transition to primary HPV screening. The aims, therefore, of the current study were: (1) to construct, calibrate and validate a complex model of HPV vaccination and cervical screening in NZ; (2) to determine, in collaboration with the NZ NCSP, a set of possible strategies (i.e. clinical management pathways) suitable to the NZ context using HPV as the primary screening test; (3) to evaluate the lifetime effects and costs associated with each of these strategies, in relation to current practice for cervical screening, in both vaccinated and unvaccinated cohorts, taking into account the levels of vaccine coverage achieved in NZ; and thus (4) to identify optimal future screening approaches in NZ in both vaccinated and unvaccinated women.

## Methods

### Construction, calibration and validation of a model of the National Cervical Screening Program in New Zealand

A dynamic model of HPV transmission and vaccination (implemented in Microsoft Visual Studio C++), coupled with a deterministic Markov model of the natural history of CIN and cervical screening and invasive cervical cancer survival (implemented using TreeAge Pro 2014, TreeAge Software, Inc., MA, USA), was used to simulate cervical disease and screening in NZ. This model platform, adaptable to different settings, has been previously used to evaluate various cervical screening and follow-up management strategies in Australia, NZ and England [10,15,16,17,18,19,20,21]. Most recently, it has been used to evaluate primary HPV screening in both unvaccinated women and cohorts offered vaccination in England [9] and in Australia [10]; the Australian findings have underpinned the recent recommendations to transition to primary HPV screening in that country.

#### Model of natural history of HPV, precancer and cervical cancer

The natural history model incorporates different disease progression and regression rates for HPV 16 infections, HPV 18 infections and other oncogenic HPV infections (HPV OHR; any oncogenic HPV type but not type 16 or 18). The dynamic HPV transmission model, adapted from previous work, [9,22,23] takes into account the effect of the National HPV Immunisation Program [3] and was used to predict HPV incidence by single year of age over time in NZ. Due to the absence of local data on HPV prevalence in the general population, the predicted prevalence of all-oncogenic and type-specific HPV infections were calibrated to that observed in Australia [24] and the UK [25]. However, the model was also calibrated to the observed age-specific rate of histologically-confirmed high-grade lesions including AGC/AIS, cervical cancer incidence and cervical cancer mortality in NZ, taking into account of the effect of cervical screening and follow-up management (see section below). The proportion for each HPV type found in HPV-positive women diagnosed with histologically-confirmed high-grade lesions [26] and cervical cancer [27] were also calibrated to local data, although we assumed that all non-misclassified high-grade precancerous lesions and all cervical cancers were causally related to HPV infection, in line with international consensus [28]. The annual progression rate from CIN3 to asymptomatic localised cancer was modelled at an age-standardised rate of 1.1%, which is consistent with the available data [29].

#### Model of current cervical screening, diagnosis and treatment in New Zealand

Current practice (CP) for cervical screening within the NCSP was modelled based on existing clinical management guidelines [1]. Adherence to screening and follow-up recommendations was modelled in detail for each management recommendation and age group, using data on cumulative attendance over time from the NZ National Cervical Screening Program Register (NCSP-R) data. The test characteristics of LBC in the local program were informed by meta-analysis [30] and calibrated to the overall and age-specific rates of each grade of cytology (ASC-US, LSIL, ASC-H and HSIL) among screened women observed locally in NZ in 2010–11; resulting in a calibrated test sensitivity of 78.6% and a specificity of 94.3% for histologically-confirmed CIN 2 or worse (CIN2+) detection at an ASC-US cut-off in women aged 20–69 years. The modelled test sensitivity and specificity varies across age-groups due to the changes in mix of the women’s underlying health state by age. More information on the age-specific test sensitivity and specificity are provided in the supplementary document S1 Appendix. The test characteristics of HPV testing as a triage test for women with low-grade cytology outcomes (ASC-US/LSIL) and as a follow-up test after treatment of CIN2/3 were derived based on the findings of a meta-analysis [31] and were further calibrated to the age-specific HPV positive rate observed in NZ in 2012–13 [32,33,34,35].

We took a health services perspective, considering screening, diagnosis and treatment costs for the NZ government. All costs are presented in NZ dollars (1 NZD = 0.7305 USD, 25 May 2015) and were inflated to 2017/18 values. The costs were obtained from the NCSP with the costs of cervical cancer treatment further calibrated to achieve an average cost of $23,116 per cancer patient as reported in 2008/09 in NZ [36]. All costs excepting the cost of HPV testing were assumed to be the same in both current practice and in primary HPV testing strategies. The unit cost of HPV test was assumed to be reduced from the current price of $43.56 to $35.00 in primary HPV screening strategies due to expected volume price breaks.

Because we have previously demonstrated in similar evaluations for Australia [10] and England [9] that the findings of evaluations of primary HPV testing are very sensitive to differing assumptions about quality-adjusted-life-year (QALY) weights, life-years (LYs) were considered as the primary outcome of the current analysis, QALYs were assessed as a secondary outcome using three sets of QALY weights—Set 1 assigned some disutility to the experience of being screened, even if the result was negative, Set 2 did not assign any disutility to the experience of being screened *per se* but assign high amount of disutility for having abnormal screening outcomes, and Set 3 did not assign any disutility to the experience of being screened *per se* but assigned a small disutility for having abnormal screening outcomes.

Further details of model structure, assumptions and the data sources are described in the supplementary document S1 Appendix.

### Determination of strategies for the evaluation

**Fig 1:**
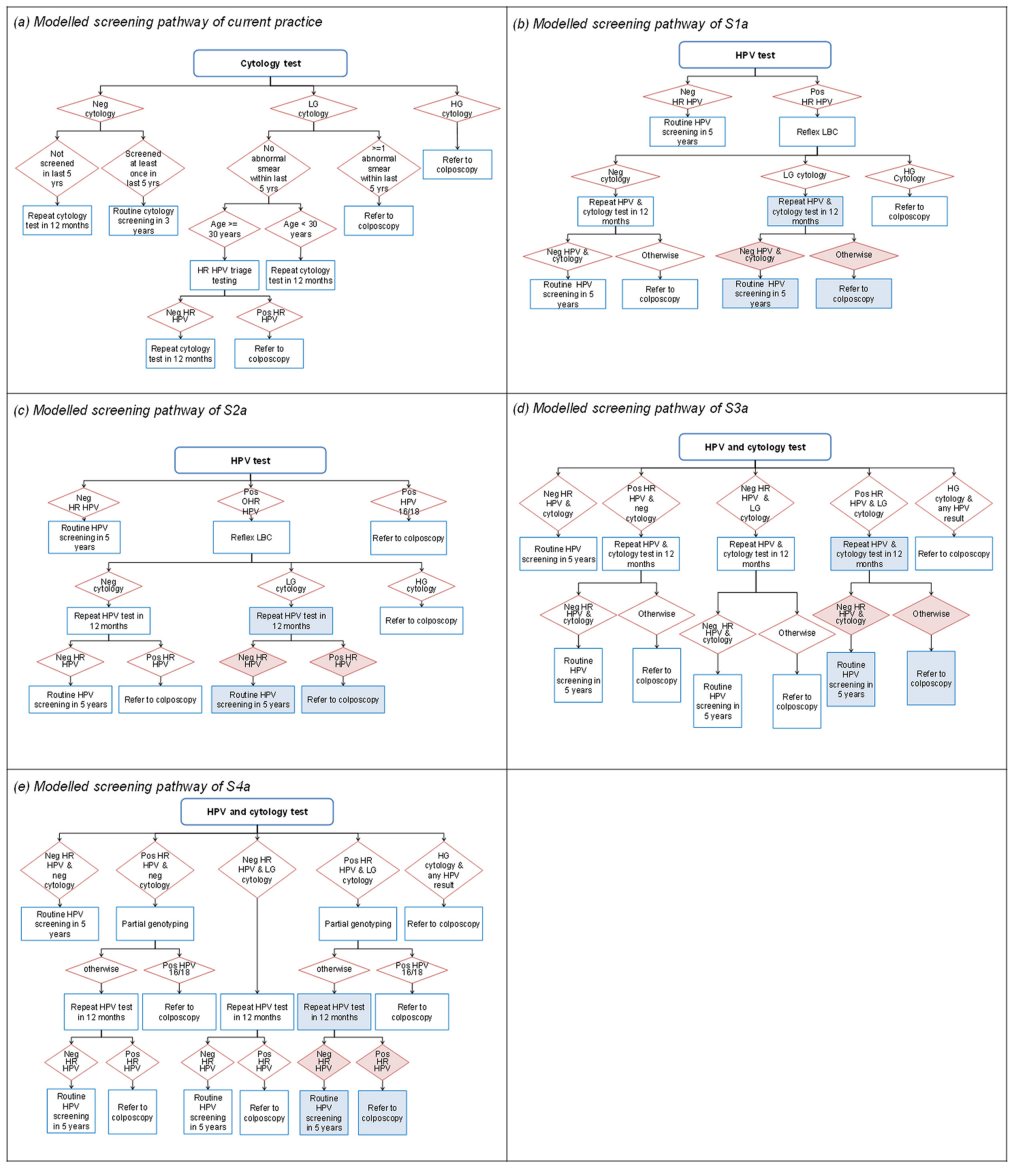
Modelled screening pathways of (a) CP, (b) S1a, (c) S2a, (d) S3a and (e) S4a. Coloured boxes indicate variations in other sub-strategies assessed. HG- High-grade (including ASC-H and HSIL); HR HPV– high risk HPV; LG –low-grade (including ASC-US and LSIL); Neg—Negative; OHR HPV- non-16/18 high -risk HPV

A total of 16 primary HPV screening strategies, determined in a series of consultations with the NCSP and its Advisory Group, were modelled (see Table 1). The strategies can be categorised into four main groups (S1-4) which varied according to the screening and triage approach (primary HPV with cytology triage for all HPV-positive; primary HPV with partial genotyping; co-testing; or co-testing with partial genotyping) and each contained four sub-strategies (a-d) which according to whether only HPV screening was used in women 25+ or whether cytology screening was used in women 20–29 years with ‘switch-over’ to HPV screening in older women, and in the management of HPV-positive women. Fig 1 shows the detailed flowcharts for current practice and primary HPV screening strategy S1a, S2a, S3a and S4a as examples. Detailed flow-charts of all screening strategies are provided in the supplementary document S1 Appendix.

**Table 1:** List of primary HPV screening strategies evaluated.

Strategy name	Age of screening starts	Screening test		Management for intermediate risk group*^,^^#^
		<30 years	30–69 years	
*Strategy 1 group*				
S1a	25	5-yearly HPV test with cytology triage	5-yearly HPV test with cytology triage	Follow-up with co-testing in 12 months
S1b	25	5-yearly HPV test with cytology triage	5-yearly HPV test with cytology triage	Immediate colposcopy
S1c	20	3-yearly cytology screening	5-yearly HPV test with cytology triage	Follow-up with co-testing in 12 months
S1d	20	3-yearly cytology screening	5-yearly HPV test with cytology triage	Immediate colposcopy
*Strategy 2 group*				
S2a	25	5-yearly HPV testing with partial genotyping & cytology triage	5-yearly HPV testing with partial genotyping & cytology triage	Follow-up with HPV testing alone in 12 months
S2b	25	5-yearly HPV testing with partial genotyping & cytology triage	5-yearly HPV testing with partial genotyping & cytology triage	Immediate colposcopy
S2c	20	3-yearly cytology screening	5-yearly HPV testing with partial genotyping & cytology triage	Follow-up with HPV testing alone in 12 months
S2d	20	3-yearly cytology screening	5-yearly HPV testing with partial genotyping & cytology triage	Immediate colposcopy
*Strategy 3 group*				
S3a	25	5-yearly co-testing with cytology & HPV test	5-yearly co-testing with cytology & HPV test	Follow-up with co-testing in 12 months
S3b	25	5-yearly co-testing with cytology & HPV test	5-yearly co-testing with cytology & HPV test	Immediate colposcopy
S3c	20	3-yearly cytology screening	5-yearly co-testing with cytology & HPV test	Follow-up with co-testing in 12 months
S3d	20	3-yearly cytology screening	5-yearly co-testing with cytology & HPV test	Immediate colposcopy
*Strategy 4 group*				
S4a	25	5-yearly co-testing with cytology & HPV test with partial genotyping	5-yearly co-testing with cytology & HPV test with partial genotyping	Follow-up with HPV testing alone in 12 months
S4b	25	5-yearly co-testing with cytology & HPV test with partial genotyping	5-yearly co-testing with cytology & HPV test with partial genotyping	Immediate colposcopy
S4c	20	3-yearly cytology screening	5-yearly co-testing with cytology & HPV test with partial genotyping	Follow-up with HPV testing alone in 12 months
S4d	20	3-yearly cytology screening	5-yearly co-testing with cytology & HPV test with partial genotyping	Immediate colposcopy

* This group refers to women who test positive for HR HPV and low-grade cytology in S1a-d and S3a-d, and women who test positive to non-16/18 HR HPV and low-grade cytology in S2a-d and S4a-d.

^#^ Women who test positive for HR HPV and negative cytology in S1a-d and S3a-d were referred to a 12 months follow-up with co-testing; women who test positive for non-16/18 HR HPV and negative cytology in S2a-d and S4a-d were referred to 12 months follow-up with HPV testing.

In the base case, primary HPV testing was assumed to have a test sensitivity of 96.4% and a specificity of 90.3% for CIN2+ detection in 20–69 years, consistent with the pooled absolute test sensitivity and specificity reported in a meta-analysis [31]. Compared to the calibrated LBC test characteristics in NZ, this has a relative sensitivity of 1.23 and a relative specificity of 0.96 for CIN2+ detection at an ASC-US+ threshold, which is consistent with the lower end of the meta-analysis findings [31], which is thus an appropriate, but favourable, assumption for cytology screening (i.e. current practice) in this evaluation.

The screening strategies were evaluated under two scenarios with respect to HPV vaccination. The ‘unvaccinated scenario’ assumed all women were unvaccinated, and thus this scenario tends to reflect outcomes in older cohorts. The ‘vaccinated scenario’ assumed women were offered HPV vaccination with uptake as observed in the cohort born in 1997 who were offered HPV vaccination at 12 years in 2009 through the HPV Immunisation Program [3] that commenced in late 2008. This cohort was selected in this evaluation on the basis that they would turn 20 years of age in 2017, and thus would be the first age group who would not be offered screening until age 25 if the current program were to change in that year. The 3-dose HPV vaccine coverage achieved in this cohort is 54% [3].

For the strategies that assumed screening starts at 25 years, in the base case we modelled a ‘rapid uptake’ at 25 years, which assumed an invitation was issued and that women currently starting screening before age 25 years would all have their first screening test at 25 years under the new recommendations. The compliance to a 5-yearly interval was derived by assuming the proportion of women have early re-screening, on-time screening and late re-screening remained similar to that observed under current practice in NZ (see supplementary document S1 Appendix for more details).

### Health, costs, and resource utilisation outcomes

The outputs of the model included cumulative lifetime risk (CLR) and age-specific rates of cervical cancer incidence and mortality, cytology test outcomes and histology test outcomes from age 10 to 84 years. Age-standardised rate (ASR) of cervical cancer incidence and mortality were calculated assuming the World Health Organisation (WHO) population.[37]. The average lifetime number of screening/follow-up episodes and colposcopy examinations were calculated by accruing the number of screening/follow-up episodes and colposcopy examinations experienced between 20 and 84 years. We defined a screening/follow-up episode to be a cytology or HPV test that a woman has for the purpose of routine screening or as follow-up after either a previous abnormal screening result or treatment for a precancerous lesion; additional cytology or HPV tests that a women may have during colposcopy or treatment for a precancerous lesion are not counted as separate screening/follow-up episodes. The number of new cervical cancer cases, cervical cancer deaths, women detected with CIN2/3, and screening, diagnostic and treatment services associated with the NCSP in 2017 were estimated assuming the 2017 NZ population [38].

### Determination of optimal strategy

The overall lifetime cost, LYs and QALYs associated with each strategy were calculated as the model output of cost, life-years and QALYs of the cohort accrued from 10 to 84 years and discounted at a rate of 3.5% p.a. from 20 years (the age of currently initiating screening). The total cost associated with the NCSP in 2017 was estimated assuming the 2017 NZ population [38]. The effectiveness and costs of each candidate strategy were considered and for strategies that were more effective than CP, we calculated the incremental cost-effectiveness ratio (ICER) compared to the next most effective strategy in order to rank the strategies in terms of their comparative cost-effectiveness in relation to each other.

### Sensitivity analysis

Univariate sensitivity analysis was performed on a list of key model assumptions listed in Table 2 to assess the robustness of the model predictions.

**Table 2:** Selected key model parameter assumptions.

Model parameters	Baseline assumption	Sensitivity analysis range	
		Min	Max
Unsatisfactory rate of cytology	1.17%	0.60%	2%
Test accuracy of cytology for primary screening, in 20–69 years ^a^^,^^b^	Sensitivity: 78.6%; Specificity: 94.3%	N/A	N/A
Test accuracy of cytology for triaging women with positive HPV result, in 20–69 years ^a^^,^^b^	Sensitivity: 78.7%; Specificity: 74.5%	N/A	Sensitivity: 84.0%; Specificity: 74.5%
Test accuracy of HPV testing for primary screening, in 20–69 years ^b^	Sensitivity: 96.4%; Specificity: 90.3%	Sensitivity: 95.1%; Specificity: 88.9%	Sensitivity: 98.6%; Specificity: 92.6%
Test accuracy of HPV testing for triaging women with ASC-US cytology result, in 20–69 years ^b^^,^^c^	Sensitivity: 90.8%; Specificity: 72.6%	Sensitivity: 89.5%; Specificity: 50.6%	Sensitivity: 94.4%; Specificity: 72.6%
Test accuracy of HPV testing for triaging women with LSIL cytology result, in 20–69 years ^b^^,^^c^	Sensitivity: 94.1%; Specificity: 47.4%	Sensitivity: 90.5%; Specificity: 24.6%	Sensitivity: 97.0%; Specificity: 47.4%
Test accuracy of HPV testing for follow-up women treated for HG CIN (TOC), in 20–69 years ^b^	Sensitivity: 92.5%; Specificity: 82.7%	Sensitivity: 85.1%; Specificity: 75.3%	Sensitivity: 96.7%; Specificity: 86.2%
Test accuracy of HPV partial genotyping among women test positive for oncogenic HPV infection	Perfect accuracy in detecting the present of HPV 16/18 infections	*(Alternative assumption 1)* 10% of women infected with HPV 16/18 will be misclassified as infected with HPV OHR	*(Alternative assumption 2)* 10% of women tested false positive against all oncogenic type will be misclassified as infected with HPV 16/18
Colposcopy positive rate	No CIN: 50.2%; CIN 1: 76.5%; CIN 2/3: 88.4%; Cancer: 100.0%	No CIN: 45.2%; CIN 1: 68.9%; CIN 2/3: 79.6%; Cancer: 100.0%	No CIN: 73.8%; CIN 1: 79.2%; CIN 2/3: 90.8%; Cancer: 100%
Screening initiation for scenario assuming screening starts from 25 years (applicable only to scenarios assumed screening starts from 25 years)	Rapid screening uptake at age of 25^d^	Gradual uptake at age of 25 years ^e^	N/A
Routine screening compliance (applicable only to scenarios assumed 5-yearly screening)	5-yearly reminder-based	5-yearly call-and-recall	N/A
Compliance to follow-up management	Base case assumption	Overall compliance rate decreased by 10%	Overall compliance rate increased by 10%
Compliance to colposcopy referral	Base case assumption	Overall compliance rate decreased by 10%	Overall compliance rate increased by 10%
Aggressiveness of CIN natural history	Base case assumption (calibrated to multiple targets)	*(5% less aggressive)* A 5% relative decrease in progression rate and increase in regression rate for all CIN transition probabilities	*(5% more aggressive)* A 5% relative increase in progression rate and decrease in regression for all CIN transition probabilities
Adjustment for ‘unmasking effect’ for HPV OHR type (application only to scenarios modelled the effect of HPV vaccination)	Base case assumption (calibrated to a ~8% increase in OHR prevalence)	No adjustment for ‘unmasking effect’	N/A
Cytology test cost	$30.19	$25.00	$35.00
HPV test cost under current practice strategies	$43.56	N/A	N/A
HPV test cost (under primary HPV screening strategies)	$35.00	$30.00	$40.00
Vaccination coverage rate	Coverage rate for female based on 3-dose data	Coverage rate for female based on 2-dose data	N/A
Discounted rate	3.5%	1.0%	5.0%

CIN: Cervical intraepithelial neoplasia; HG: high-grade; HPV OHR: oncogenic HPV type other than HPV 16 or 18; TOC: Test-of-cure;

^a^ Cut-off at ASC-US threshold

^b^ For CIN2+detection

^c^ Same test accuracy was modelled for HPV triage testing for women with both ASC-US and LSIL cytology result. Differences in the test sensitivity and specificity were due to the differences in the mix of underlying health states between women with ASC-US and LSIL cytology result.

^d^ Assume women who have had their first screening test at age < = 25 years under current practice would all have their first screening test at age 25 years in the scenario assuming screening starts from 25 years

^e^ Assume a gradual screening initiation in 25–29 years. The proportion of women who have had their first screening test by the age of 30 of the scenario was assumed to be the same as the proportion assumed for current practice.

### Ethical considerations

The National Cervical Screening Programme Register data used to inform model parameters were de-identified. The Cancer Council NSW Human Research Ethics Committee approved the transfer of these data to the researchers and the New Zealand Ministry of Health approved their use in this modelled evaluation.

## Results

### Model calibration and validation

The calibrated model outcomes showed close correspondence with observed age-specific rates of HPV prevalence [24,25], HPV type distribution in women with HPV-positive histologically-confirmed disease or cervical cancer [26,27], histologically-confirmed high-grade abnormalities, cytology outcomes [39], overall age-specific cervical cancer incidence and mortality [40,41,42,43,44,45,46], and cancer stage distribution [47]. The model outputs for overall screening participation (given the detailed age and management specific adherence assumptions used as inputs) and cytology test performance were validated against recent data observed in NZ [39]. See supplementary document S1 Appendix for more detailed model calibration and validation outcomes.

### Health, costs and cost-effectiveness outcomes

#### Health outcomes

In the NZ population of 2.3 million women aged <85 years in 2017, if current practice (CP) for screening was retained, a total of 160 new cervical cancer cases, 45 cervical cancer deaths and 4,038 women detected with histologically-confirmed high-grade were estimated to occur in the unvaccinated scenario; with 92 new cancer cases, 26 cancer deaths and 2,645 cases of histologically-confirmed high-grade disease in the vaccinated scenario (Table 3). The predicted ASRs for cervical cancer incidence and mortality were 9.1 and 1.5 per 100,000 women aged 20–69 years, respectively in the unvaccinated scenario, and 5.2 and 0.8, respectively in the vaccinated scenario. The predicted CLRs of cervical cancer incidence and of cervical cancer mortality were 0.63% and 0.20%, respectively in the unvaccinated scenario, and 0.36% and 0.12%, respectively in the vaccinated scenario.

**Table 3:** Model-predicted cervical cancer incidence, cervical cancer death, histologically-confirmed CIN2/3 and cost associated with screening program.

Strategies	Cervical cancer incidence				Cervical cancer death				Number of CIN2/3 detected ^b^	Total cost associated with screening program ^a^^,^^b^	
	CLR^a^	Cases ^a^^,^^b^	ASR ^c^	*% change in ASR* ^d^	CLR^a^	Cases ^a^^,^^b^	ASR ^c^	*% change in ASR* ^d^		Cost (in $M)	Difference compare to CP (% change)
*Unvaccinated scenario*											
***CP***	***0*.*63%***	***160***	***9*.*1***	***-***	***0*.*20%***	***45***	***1*.*5***	***-***	***4*,*038***	***$31*.*7 M***	***-***
S1a	0.64%	161	9.1	*0*.*1%*	0.20%	45	1.5	*0*.*3%*	3,704	$28.7 M	*-$3*.*0 M (-9%)*
S1b	0.57%	144	8.1	*-11*.*1%*	0.18%	40	1.3	*-11*.*9%*	3,998	$31.1 M	*-$0*.*6 M (-2%)*
S1c	0.66%	167	9.4	*3*.*3%*	0.21%	48	1.6	*5*.*0%*	3,962	$29.3 M	*-$2*.*4 M (-8%)*
S1d	0.62%	157	8.9	*-2*.*1%*	0.20%	44	1.5	*-1*.*9%*	4,056	$30.5 M	*-$1*.*1 M (-4%)*
S2a	0.56%	140	7.7	*-15*.*2%*	0.18%	39	1.3	*-15*.*6%*	3,995	$30.4 M	*-$1*.*3 M (-4%)*
S2b	0.54%	136	7.5	*-17*.*3%*	0.17%	38	1.2	*-18*.*0%*	4,118	$32.2 M	*$0*.*5 M (2%)*
S2c	0.62%	157	8.9	*-2*.*1%*	0.20%	44	1.5	*-2*.*1%*	4,012	$29.5 M	*-$2*.*1 M (-7%)*
S2d	0.60%	154	8.7	*-3*.*7%*	0.19%	43	1.4	*-4*.*0%*	4,069	$30.7 M	*-$1*.*0 M (-3%)*
S3a	0.63%	158	8.9	*-2*.*1%*	0.20%	45	1.5	*-1*.*3%*	3,785	$36.4 M	*$4*.*7 M (15%)*
S3b	0.57%	143	7.9	*-12*.*6%*	0.18%	40	1.3	*-12*.*6%*	4,069	$38.7 M	*$7*.*0 M (22%)*
S3c	0.65%	165	9.3	*2*.*0%*	0.21%	47	1.6	*3*.*9%*	3,992	$35.5 M	*$3*.*8 M (12%)*
S3d	0.61%	155	8.8	*-3*.*1%*	0.20%	44	1.5	*-2*.*5%*	4,082	$36.7 M	*$5*.*0 M (16%)*
S4a	0.54%	135	7.5	*-17*.*6%*	0.17%	38	1.2	*-17*.*7%*	4,073	$38.3 M	*$6*.*6 M (21%)*
S4b	0.53%	132	7.3	*-19*.*5%*	0.17%	37	1.2	*-19*.*9%*	4,191	$40.0 M	*$8*.*4 M (26%)*
S4c	0.60%	152	8.7	*-4*.*6%*	0.19%	43	1.4	*-4*.*6%*	4,055	$35.9 M	*$4*.*3 M (13%)*
S4d	0.59%	150	8.5	*-6*.*1%*	0.19%	42	1.4	*-6*.*4%*	4,109	$37.1 M	*$5*.*4 M (17%)*
*Vaccinated scenario*											
***CP***	***0*.*36%***	***92***	***5*.*2***	***-***	***0*.*12%***	***26***	***0*.*8***	***-***	***2*,*645***	$25.9 M	*-*
S1a	0.37%	93	5.2	*0*.*2%*	0.12%	26	0.9	*0*.*3%*	2,401	$22.5 M	*-$3*.*5 M (-13%)*
S1b	0.33%	83	4.6	*-10*.*9%*	0.10%	23	0.7	*-11*.*8%*	2,616	$24.6 M	*-$1*.*3 M (-5%)*
S1c	0.38%	96	5.4	*3*.*5%*	0.12%	27	0.9	*5*.*3%*	2,579	$23.3 M	*-$2*.*7 M (-10%)*
S1d	0.35%	90	5.1	*-2*.*2%*	0.11%	25	0.8	*-2*.*0%*	2,655	$24.5 M	*-$1*.*5 M (-6%)*
S2a	0.33%	83	4.6	*-11*.*7%*	0.11%	23	0.7	*-11*.*9%*	2,527	$22.7 M	*-$3*.*2 M (-12%)*
S2b	0.32%	79	4.4	*-15*.*7%*	0.10%	22	0.7	*-16*.*5%*	2,667	$24.7 M	*-$1*.*2 M (-5%)*
S2c	0.36%	92	5.2	*-0*.*3%*	0.12%	26	0.9	*0*.*3%*	2,595	$23.0 M	*-$3*.*0 M (-11%)*
S2d	0.35%	89	5	*-3*.*3%*	0.11%	25	0.8	*-3*.*4%*	2,657	$24.3 M	*-$1*.*7 M (-6%)*
S3a	0.36%	91	5.1	*-2*.*1%*	0.12%	26	0.8	*-1*.*4%*	2,463	$30.3 M	*$4*.*3 M (17%)*
S3b	0.33%	82	4.5	*-12*.*5%*	0.10%	23	0.7	*-12*.*6%*	2,672	$32.3 M	*$6*.*4 M (25%)*
S3c	0.38%	95	5.3	*2*.*1%*	0.12%	27	0.9	*4*.*1%*	2,604	$29.5 M	*$3*.*6 M (14%)*
S3d	0.35%	89	5	*-3*.*3%*	0.11%	25	0.8	*-2*.*7%*	2,677	$30.7 M	*$4*.*8 M (18%)*
S4a	0.32%	81	4.5	*-14*.*1%*	0.10%	23	0.7	*-13*.*9%*	2,587	$30.6 M	*$4*.*7 M (18%)*
S4b	0.31%	77	4.3	*-17*.*8%*	0.10%	21	0.7	*-18*.*2%*	2,723	$32.5 M	*$6*.*6 M (25%)*
S4c	0.35%	90	5.1	*-2*.*7%*	0.11%	25	0.8	*-2*.*0%*	2,626	$29.4 M	*$3*.*4 M (13%)*
S4d	0.34%	87	4.9	-5.4%	0.11%	24	0.8	*-5*.*5%*	2,686	$30.6 M	*$4*.*6 M (18%)*

ASR- age-standardised rate; CLR- cumulative lifetime risk;

^a^ 0–84 years

^b^ Assuming 2017 New Zealand female population

^c^ Rate per 100,000 women in 20–69 years, assuming WHO population

^d^ Compared to value predicted for CP

The relative effectiveness of each primary HPV screening strategy in relation to CP was similar in both unvaccinated and vaccinated scenarios. Twelve out of the sixteen primary HPV strategies were predicted to be associated with a decrease (of 2–20%) in cervical cancer incidence and mortality. Strategies varied in terms of their impact on the number histologically-confirmed high-grade cases, ranging from a +/-7% difference compared to CP (Table 3).

Partial genotyping strategies (S2 and S4 strategy groups) were associated with a 1–16% relative decrease (unvaccinated scenario: 2–16%; vaccinated scenario:1–13%) in cancer incidence and mortality when compared to the equivalent non-partial genotyping strategies (S1 and S3 strategy groups); HPV and cytology co-testing strategies (S3 and S4 strategy groups) were associated with a modest (<1–3%) relative decrease in both scenarios when compared to the equivalent non-co-testing strategies (S1 and S2 strategy groups).

Within each strategy group, strategies assuming primary HPV screening starting at age 25 years (strategy sub-groups a and b) were predicted to be associated with a 3–15% relative decrease in cancer incidence and mortality when compared to the equivalent ‘switch-over’ strategy (strategy sub-groups c and d); strategies that refer ‘intermediate risk’ women to immediate colposcopy (strategy sub-groups b and d) were predicted to be associated with a 2–12% relative reduction in both scenarios when compared to the equivalent strategies that referring ‘intermediate risk’ women to 12 months follow-up (strategy sub-groups a and c). However, this difference was minimised in partial genotyping strategies, where the relative difference was only 2–5% (since the highest risk HPV16/18 positive women are referred directly to colposcopy in such strategies), compared to 5–12% in strategies without partial genotyping.

**Fig 2:**
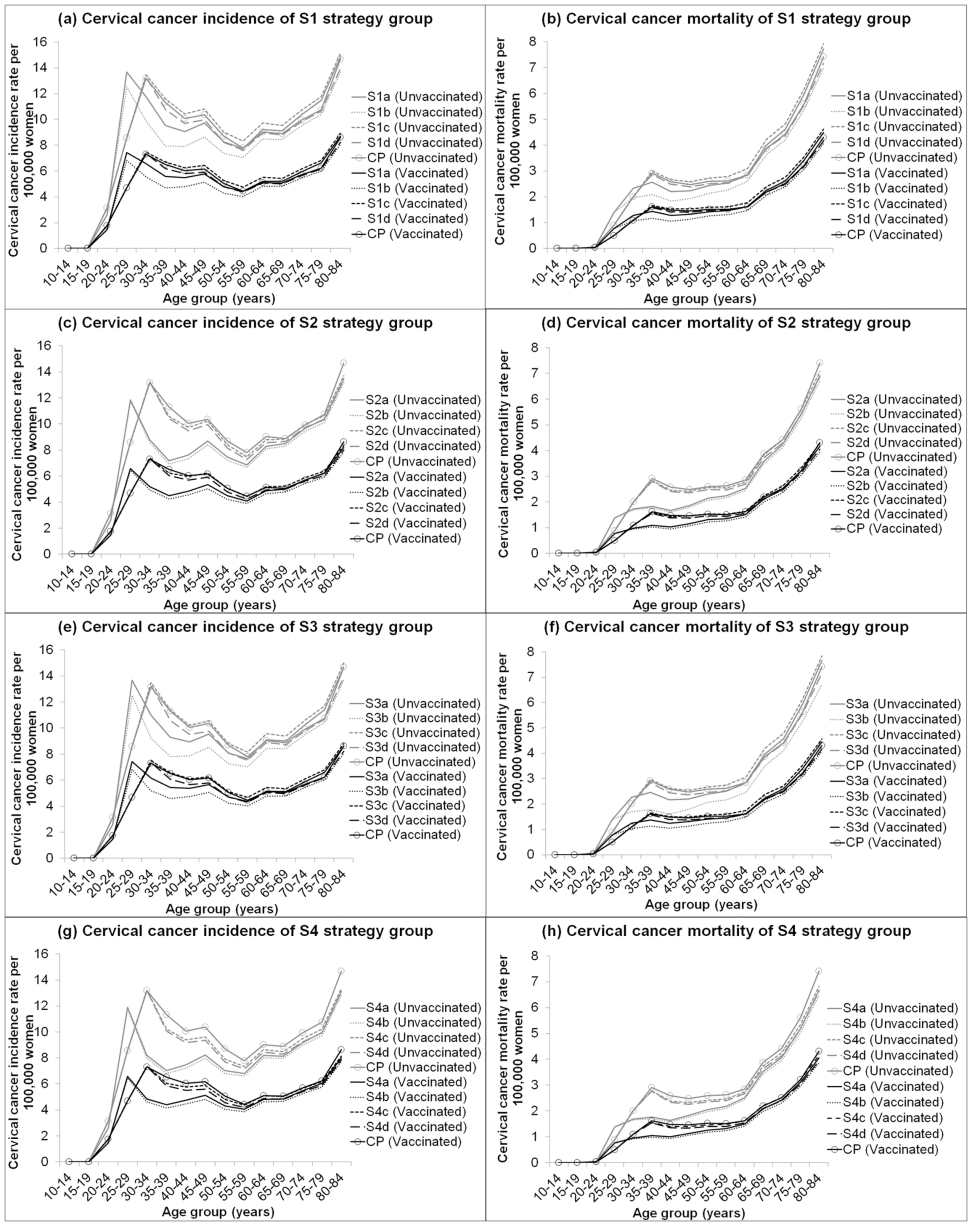
Estimated age-specific of cervical cancer incidence rate and cervical cancer mortality rate for all strategies.

Compared to CP, starting screening at 25 years with primary HPV testing (strategy sub-groups a and b) was predicted to be associated with a higher cancer incidence rate in 25–29 year-old women (the first 5 years after screening begins) but a significantly lower rate in 30+ years (Fig 2). Partial genotyping strategies were estimated to have a lower cervical cancer incidence and mortality rates in all ages compared to strategies that do not employed partial genotyping. The ‘switch-over’ strategies were estimated to have a similar rate to CP at all ages.

#### Costs

Screening according to current practice is predicted to cost $31.7M to the NZ government in 2017 (unvaccinated) and $25.9M (vaccinated) (Table 3). This includes cervical screening, follow-up, and treatment for precancerous lesions and cancer. Compared to CP, primary HPV with cytology triage (S1 and S2) were predicted to be associated with a 3–12% decrease in costs but HPV and cytology co-testing strategies (S3 and S4) were predicted to be associated with a 12–26% increase in costs.

#### Cost-effectiveness

**Fig 3:**
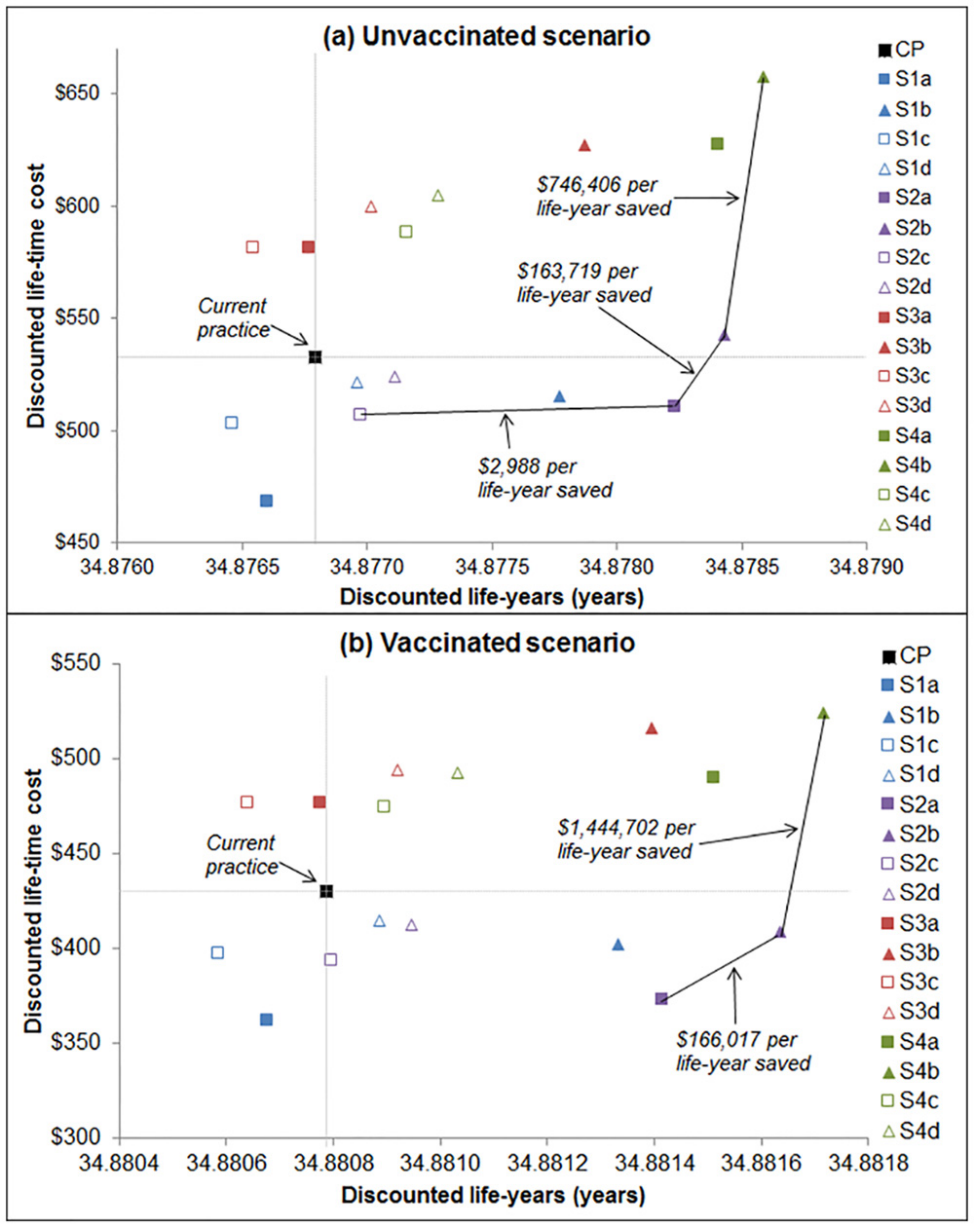
Cost-effectiveness planes for (a) unvaccinated scenario and (b) vaccinated scenario.

All strategies assuming primary HPV testing (S1 and S2 groups), were more effective than CP, and all except S2b were also cost-saving. All co-testing strategies (S3 and S4 groups) were both more effective and more costly than CP. Fig 3 shows the cost-effectiveness planes for the unvaccinated and the vaccinated scenario. We could not follow usual practice for deriving ICERs referenced to CP as the comparator, since some of the new strategies considered were both more effective and more cost-saving than CP. Therefore, for the current analysis, we did not consider CP as the comparator and included only the strategies that were more effective than CP. In this modified ICER analysis (which thus requires careful interpretation), S2a was the only strategy that was associated with an ICER that was below the $20,000-$50,000 per life-year saved indicative willingness-to-pay threshold for NZ in the analysis in both scenarios. This strategy is also a cost-saving strategy (more effective and less costly) when compared to current practice.

Supplementary cost-effectiveness analyses were undertaken using health utilities to incorporate the effect on morbidity as well as mortality. Findings varied widely with the different utility sets. Strategy S2a was predicted as the most cost-effective (and cost-saving) strategy in both the unvaccinated and vaccinated scenarios when QALY weights set 1 and 3 were assumed; all primary HPV screening strategies were predicted to be less effective than current practice when QALY weights set 2 was assumed. Detailed outcomes of the supplementary analysis are provided in the supplementary document S1 Appendix.

### Resource utilisation

**Fig 4:**
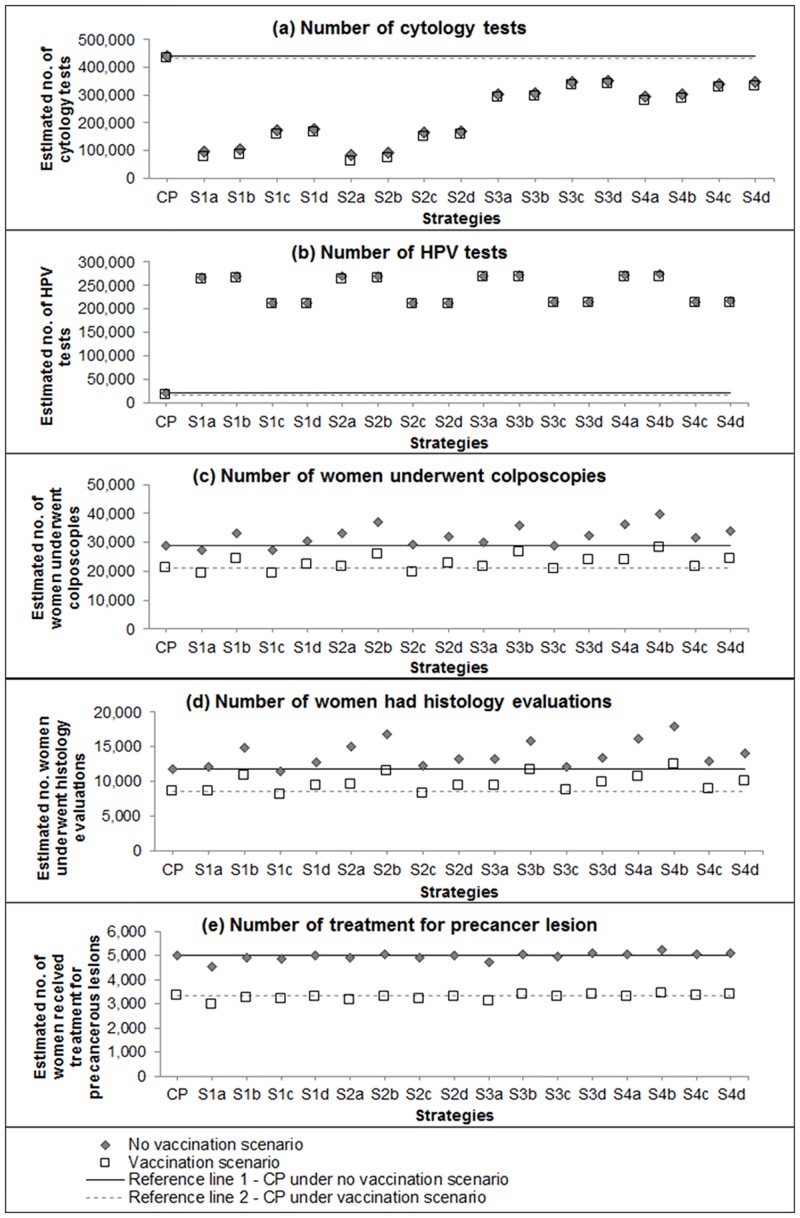
Model-predicted resource utilisation for all, assuming 2017 Australian female population.

Fig 4a–4e shows the estimated number of cytology tests, HPV tests, women undergoing colposcopy examination, women having histology and women having treatment for precancerous lesion, assuming the female population predicted for NZ in 2017. Under CP management, approximately 28,800 women were estimated to undergo colposcopy examination and 11,800 women to have at least one histology evaluation in the unvaccinated scenario; in the vaccinated scenario, the numbers reduced to 21,000 and 8,600, respectively. Many of the primary HPV screening strategies were predicted to be associated with an increase in number of colposcopy examinations and histology evaluations in the unvaccinated scenario but in the vaccinated population the partial genotyping strategy S2a would not result in any substantial change in colposcopies.

About 5,020 women in the unvaccinated scenario and 3,350 women in vaccinated scenario were estimated to undergo treatment for precancer under CP; most primary HPV screening strategies would result in a broadly similar number of women undergoing treatment (unvaccinated scenario: range 4,560–5,220; vaccinated scenario: range 2,980–3,440).

### Lifetime numbers of screening/follow-up episodes and colposcopies

**Fig 5:**
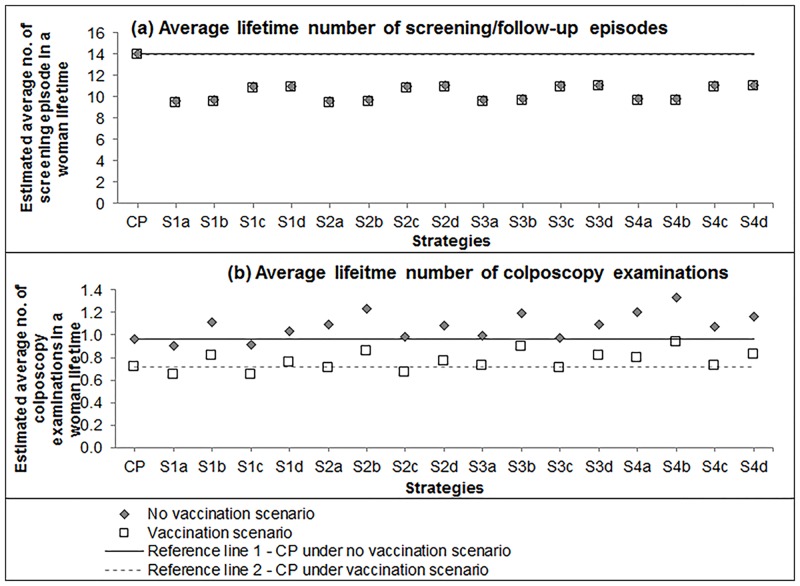
Model predicted average lifetime number of (a) screening/follow-up episodes and (b) number of colposcopies examinations.

Under CP screening management, given the adherence assumptions used, a woman was predicted to experience 14.1 screening and/or follow-up episodes on average in her lifetime in the unvaccinated scenario and 14.0 episodes in the vaccinated scenario. This number will reduce to an average of 9.7 (unvaccinated scenario: 9.6–9.9; vaccinated scenarios: 9.4–9.7) episodes for primary HPV screening strategies screening starting from age 25 years (sub-strategies a and b), and to an average of 11.0 (unvaccinated: 11.0–11.2 episodes; unvaccinated scenario: 10.8–11.0) episodes for the ‘switch-over’ strategies (sub-strategies c and d) (Fig 5). On average, a woman was estimated underwent 1.0 colposcopy examination in her lifetime in the unvaccinated scenario and 0.7 examinations in the vaccination scenarios under CP screening management. The number increased in most of primary HPV screening strategies (unvaccinated scenario: 1.0–1.3; vaccinated scenarios: 0.7–0.9).

### Number of precancer treatments per cervical cancer case prevented

**Fig 6:**
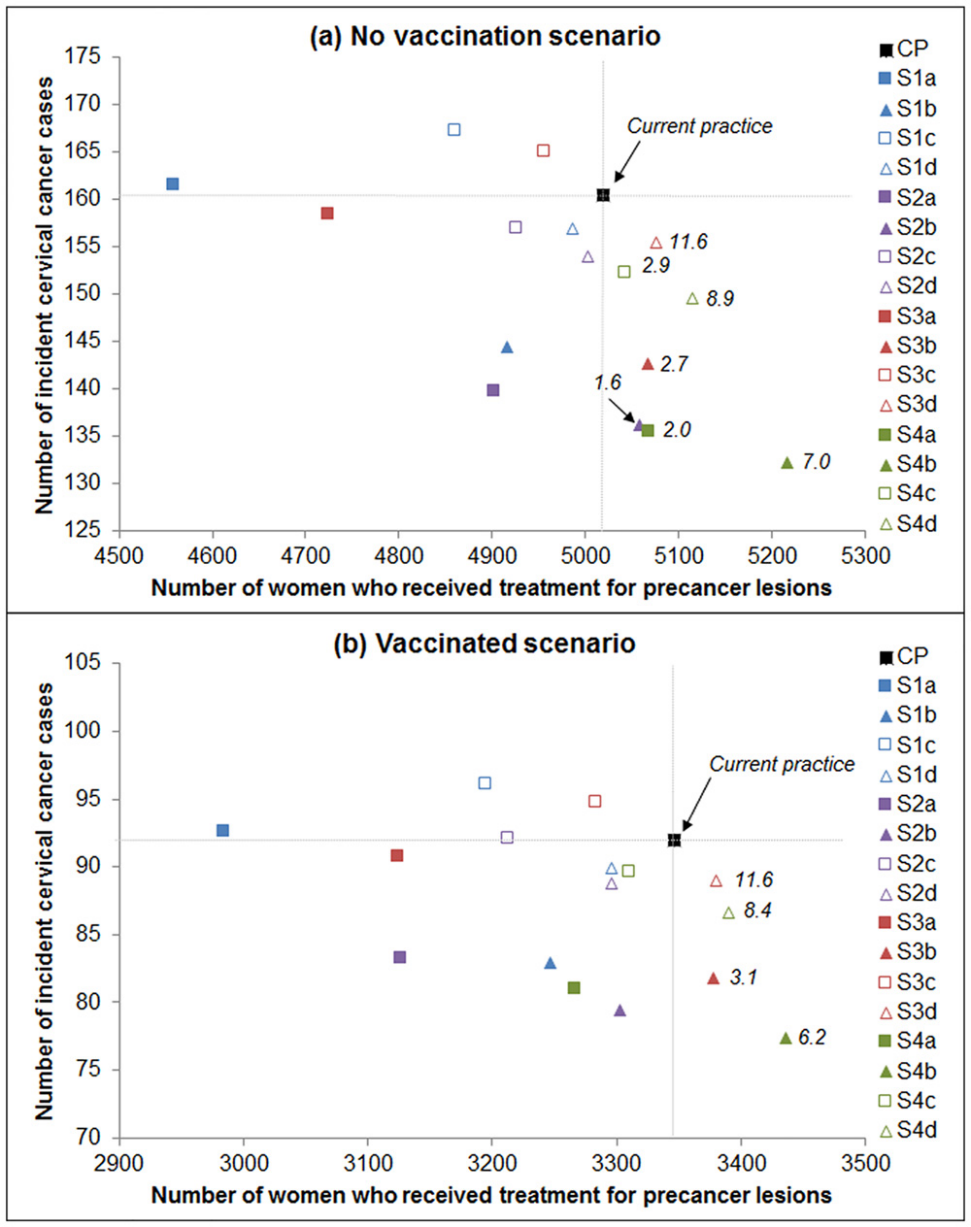
Comparison of the number of cervical cancer cases with the number of women who received treatment for precancer predicted for each strategy. Numbers shown on the chart represent the number of additional treatments required per cancer case prevented compared to current practice. The number of additional treatments required per cancer case prevented ratio was calculated for each strategy with a higher number precancer treatments and lower number of cervical cancer cases than CP. The calculated ratio is display in the figure on side of the marker that represents the strategy.

Fig 6 compares the number of cervical cancer cases with the number of women who received treatment for precancer predicted for each strategy. There are six primary HPV screening strategies in the no vaccination scenario and eight strategies in the vaccinated scenario predicted to be associated with fewer cervical cancer cases and fewer women treated for precancer lesions than CP. Seven strategies in the no vaccination scenario and four strategies in the vaccinated scenario were associated with fewer cervical cancer cases but more of women treated for precancer lesions than CP. Among these scenarios, the number of additional treatments required per cancer case prevented ranged from 1.6–11.6 and 3.1–11.6, respectively (Fig 6).

### Sensitivity analysis

Univariate sensitivity analysis was conducted for S2a and S2c in both unvaccinated and vaccinated scenarios. The predicted costs were found to be sensitive to the assumptions made for the cytology test cost, the screening initiation patterns, test characteristics of HPV test for primary screening or for triage testing on women with low-grade cytology results (which changes the predicted comparator outcomes), the cost of HPV testing, the accuracy of specific HPV partial genotyping outcomes and the aggressiveness of natural history in both scenarios. For S2a, the predicted life-years were found to be sensitive to the assumptions made for the aggressiveness of natural history (although this has been extensively calibrated over time for the model platform), and adherence to the recommendation to initiate screening at age 25 years (i.e. for most women to initiate screening at or close to that age and not later). Increased test sensitivity for both LBC as a triage test for women with a positive HPV test result and HPV as a triage test for women with low-grade cytology outcome were found to have minimal impact on the cost and life-years estimated for S2a. The detailed outcomes of the sensitivity analysis are provided in the supplementary document S1 Appendix.

## Discussion

We have performed a comprehensive health and economic evaluation of primary HPV testing for cervical cancer screening in New Zealand, which takes into account the impact of a national HPV vaccination program in that country. In this analysis, we found broad similarities in the relative outcomes between alternative screening strategies when considering unvaccinated women and women offered vaccination. Most (but not all) of the primary HPV screening strategies we considered were predicted to be more effective in reducing cancer incidence and mortality compared to current screening practice. Many of the primary HPV screening strategies, with the exception of co-testing, were also predicted to be less costly than current practice. In the cost-effectiveness analysis, primary screening with partial genotyping for women aged 25–69 years, with direct referral of HPV16/18 positive women to colposcopy and LBC triage for women positive for other oncogenic types with 12 month follow-up for LBC negative and low-grade results in that group (Strategy S2a) was identified as both more effective and also cost saving compared to current practice, and in comparative incremental analysis was the most cost-effective strategy in both scenarios. This strategy was predicted to be associated with a further 12–16% reduction in cervical cancer incidence and mortality and a saving of $NZ1.3 M—$3.2 M (4–12%) of the total program cost of screening compared to current practice. A variation of this strategy, where women testing positive for other (non-HPV16/18) oncogenic types but who had low-grade cytology were referred directly for colposcopy (Strategy S2b), was marginally more effective, but not cost-effective (>$160,000/LYS) compared to a $20,000-$50,000 per life-year saved indicative willingness-to-pay threshold in New Zealand (where the upper end reflects the approximate GDP per capita in March 2015).

The three QALY weight sets assessed in the supplementary analysis of this study were obtained from three different studies [48,49,50] and the outcome varied significantly when different sets were assumed. Similar to the findings of previous evaluations [9,10], we found that the model’s findings are very sensitive to differing assumptions about QALY weights. Therefore, life-years were considered as the primary outcome of the current analysis and QALYs were assessed as a secondary outcome. The QALY-related findings thus need to be interpreted keeping in mind the significant variation in QALY weights found by different studies.[48,49,50] More study on the health utilities related to cervical screening is required; focus groups or other methods could be used to perform more detailed assessments of the quality of life utilities associated with the experience of having a cervical sample taken (and waiting for the result), of having an abnormal cytology test or positive HPV test, and of being referred for colposcopy and having treatment and post-treatment surveillance.

The current evaluation was performed on behalf of the National Cervical Screening Programme (NCSP) and overseen by the programme’s Advisory Group. This was done in the context of the recent announcement by the Australian government that it will transition to 5-yearly HPV screening in women aged 25–70 years by May 2017; the effectiveness modelling and economic evaluation to support that decision in Australia harnessed the same modelled platform and methods as for the current evaluation in New Zealand [10]. As for our prior evaluation in Australia, we found that the optimal strategy for primary HPV screening strategies would be *both* more effective and cost saving when compared to current screening practice, reducing cervical cancer incidence and mortality in New Zealand by a further 12–16% compared to current levels. A strength of the evaluation is that it used a comprehensive and calibrated model of cervical screening, based on detailed clinical management guideline recommendations, taking into account the observed screening behaviour. We conducted detailed evaluations of a wide range of primary HPV testing management options and these findings will inform policy makers of the potential benefit as well as the impact on resource utilisation associated with primary HPV testing in NZ.

A limitation of this study was that a number of influential parameters related to future screening practices, by necessity, were based on assumptions. However, these assumptions had been extensively discussed with in-country experts at the NCSP. For the primary HPV testing strategies, we assumed ~80% of women will attend for their first screening test at 25 years (based on the current uptake rate observed in women aged < = 25 years) and that the compliance rate to a 5-year screening interval recommendation will be consistent with that currently observed for the recommended 3-year interval, including limited early re-screening. After consultation, we also assumed the cost of HPV test would reduce to $35 per test when HPV test was used as the primary screening test (assuming a lower unit test cost could be negotiated with a larger volume of tests being purchased). The sensitivity analysis has found that the cost outcomes are sensitive to this assumption and thus this is a prime consideration for decision-makers in the transition to primary HPV screening. However, the optimal strategy for HPV screening with partial genotyping is still predicted to be cost saving compare to current practice when assuming HPV test cost was $40 per test in sensitivity analysis.

This evaluation was conducted using assumptions that were favourable for cytology testing and conservative with respect to primary HPV testing. Specifically, the base case assumptions for HPV test characteristics were derived such that the relative test sensitivity and specificity (compared to base case cytology test characteristics) was consistent with the lower end of the 95%CI of the findings of a major meta-analysis [31]–that is, assumed the minimum improvement in the screening test sensitivity and maximum loss in test specificity when HPV test was used for screening compared to cytology. Additionally, the potential degradation in cytology performance due either to HPV 16/18 depletion (reduced cytological abnormalities) and its subsequent impact on cytology accuracy (reduced PPV for CIN2/3) or cytologist de-training effects [48] were not accounted for in the vaccinated scenario in this evaluation. Furthermore, the differential test accuracy for glandular lesions of cytology and HPV test, which is expected to be relatively higher for HPV testing, was not modelled in this evaluation. Finally, the modelled cost of HPV test under primary HPV screening strategies ($NZ 35.00 per test) was conservatively assumed to be modestly higher than the cost cytology test ($NZ 30.19) based on the advice of the NCSP. The modelled strategies would be associated with a greater cost-saving if the cost of the HPV test was lower than the current cost of cytology test. Therefore, our favourable findings with respect to primary HPV screening can be considered to be based on conservative ‘reasonable worst case’ assumptions for HPV screening effectiveness.

We found that most of the primary HPV screening strategies were predicted to increase the number of women requiring access to diagnostic services, if the impact of vaccination is not considered. This finding implies that the colposcopy referral and histology evaluation rates may increase initially after a transition to primary HPV screening but are expected to drop over time as cohorts offered vaccination age and enter the new HPV screening program. However, it should be noted that the current estimates represent ‘steady state’ outcomes and transitional fluctuations in test volumes and referral rates would be expected after the transition of the screening program.

We found that 5-yearly primary HPV screening would be more effective and cost-saving compared to the current 3-yearly cytology screening program in NZ, and that managing women based on HPV partial genotyping was a highly effective, and the most cost-effective, option. We also found that co-testing with cytology is not cost-effective. These findings are consistent with the results of similar modelled evaluations conducted for England and Australia [9,10].

It is notable that, although NZ and Australia have differing recommendations for current practice for cervical cytology screening (i.e. 3-yearly screening in NZ vs. 2- yearly in Australia), the optimal strategy identified in this evaluation for NZ (HPV testing with partial genotyping for HPV16/18 and LBC triage for OHR HPV types) is very similar to the proposed pathway for primary HPV screening identified in the National Cervical Screening Program Renewal for Australia [49]. It also closely corresponds to one arm of the Compass trial, a large scale randomised controlled trial of primary HPV screening commenced in Australia in 2013 (ACTRN12613001207707) and the main trial of 121,100 women has begun in 2014 (NCT02328872); the trial was extended to NZ in 2014.

The assessed primary HPV screening strategies were determined in a series of consultations with the NCSP and its Advisory Group, and were modelled in the context of New Zealand, which has an effective cervical screening program that provides 3-yearly routine screening using cytology test with a high test sensitivity (~79% for CIN2+ detection) for 20–69 years. Although the optimal screening strategy identified by this study is also similar to the proposed pathway identified in the National Cervical Screening Program Renewal for Australia[49], some of the study specific outcomes may not be applied directly to some other country setting. For example, 5-yearly primary HPV screening may be associated with a greater health benefit gained but not cost saving in countries with cytology screening programs that recommend less frequent screening (i.e. 5-yearly) or which use a cytology test with lower test sensitivity; countries with cytology screening programs that recommend more frequent screening (i.e. annual or biennial) or which use a cytology test with higher test sensitivity may find a lesser health benefit gained associated with 5-yearly primary HPV testing. The acceptability of the various strategies, and in particular the management of women with non-16/18 HPV positive and low-grade cytology result is likely to depend not only evidence generated from carefully conducted modelled evaluations but also to some extent on country specific issues and considerations which include the ‘starting point’ i.e. current acceptable management in different situations.

NZ had a challenging history with respect to the original introduction and development of cervical screening [50,51]. Therefore, changes to the program will involve wide consultation with women and providers. The NZ service evaluation experience represented by the extension of *Compass* will play a role in this process- *Compass NZ* (ACTRN12614000714684) has recruited five hundred 25–64 year-old women presenting for cervical screening for 5-yearly HPV screening with partial genotyping for HPV 16/18, to assess feasibility of this approach via participant acceptability, promoting GP, nurse and colposcopist education, and facilitating laboratory implementation. Since *Compass NZ* utilises the same pathway for primary HPV screening as that identified in this modelled analysis as the optimal approach, it represents a practical experience of primary HPV screening with this pathway before its implementation in NZ.

## Conclusion

We predict that primary HPV screening with genotyping for HPV16/18 will be both more effective and less costly than the current program for 3-yearly cytology-based screening in NZ, in both unvaccinated women and in cohorts offered vaccination. These findings are in accordance with modelled evaluations for Australia and England and are consistent with the data from large scale international trials of primary HPV screening. Any change in national policy will involve extensive public consultation and will take into consideration broader criteria beyond cost-effectiveness.

## Supporting Information

S1 Appendix Supplementary materials. (DOCX)

## Abbreviations

AGC: Atypical glandular cells
AIS: Adenocarcinoma in situ
ASC-H: Atypical squamous cells-cannot exclude high-grade squamous intraepithelial lesion
ASC-US: Atypical cells of undetermined significance
ASR: Age-standardised rate
CIN 2/3: Histologically-confirmed high-grade cervical intraepithelial neoplasia grade 2 or 3
CIN2+: Histologically-confirmed high-grade cervical intraepithelial neoplasia grade 2 or worse
CLR: Cumulative lifetime risk
CP: Current practice
HPV: Human papillomavirus
HPV OHR: any oncogenic HPV type but not type 16 or 18
HSIL: High-grade squamous intraepithelial lesion
ICER: Incremental cost-effectiveness ratio
LSIL: Low-grade squamous intraepithelial lesion
LY: life-year
NCSP: National Cervical Screening Program
NCSP-R: National Cervical Screening Program Register
NZ: New Zealand
QALY: Quality adjusted life-year
