# Supplementary material for: Effectiveness Modelling and Economic Evaluation of Primary HPV Screening for Cervical Cancer Prevention in New Zealand
Source: PLoS One. 2016 May 17;11(5):e0151619. doi: 10.1371/journal.pone.0151619 (PMC4871332; doi:10.1371/journal.pone.0151619)
Supplement: S1 Appendix — (DOCX) [file pone.0151619.s001.docx]

Supplementary Material S1 Appendix accompanying the article:

**Effectiveness Modelling and Economic Evaluation of Primary HPV Screening for Cervical Cancer Prevention in NZ**

Jie-Bin Lew,^1§^ Kate Simms,^1^ Megan Smith,^1^ Hazel Lewis,^2^ Harold Neal,^3^ and Karen Canfell^1,4^

^1^ Cancer Research Division, Cancer Council NSW, New South Wales, Australia.

^2^ Public Health Physician, Wellington, New Zealand.

^3^ National Screening Unit, Ministry of Health, Wellington, New Zealand.

^4^ School of Public Health, Sydney Medical School, University of Sydney, Australia.

^§^ Corresponding author.

Contents

[1 OVERVIEW OF MODEL PLATFORM 4](#_Toc448766802)

[2 STRATEGIES FLOW CHARTS 7](#_Toc448766803)

[2.1 Current practice 7](#_Toc448766804)

[2.2 Primary HPV testing strategies 8](#_Toc448766805)

[2.3 Follow-up in women with abnormal screening outcome 17](#_Toc448766806)

[2.4 Follow-up management for women treated for high-grade CIN 21](#_Toc448766807)

[3 DATA SOURCES 25](#_Toc448766808)

[3.1 HPV incidence and CIN natural history model 25](#_Toc448766809)

[3.2 Cancer survival 25](#_Toc448766810)

[3.3 Mortality and hysterectomy rate 25](#_Toc448766811)

[3.4 Screening and diagnostic test characteristics and data sources 26](#_Toc448766812)

[3.4.1 Liquid-based cytology (LBC) test 26](#_Toc448766813)

[3.4.2 HPV test for primary screening 30](#_Toc448766814)

[3.4.3 Triage HPV test for low-grade cytology 32](#_Toc448766815)

[3.4.4 HPV test for follow-up women treated for high-grade lesion 35](#_Toc448766816)

[3.4.5 Colposcopy 36](#_Toc448766817)

[3.5 Screening initiation assumptions 37](#_Toc448766818)

[3.6 Screening compliance assumptions 39](#_Toc448766819)

[3.6.1 Routine screening at 3-yearly interval (Current practice) 39](#_Toc448766820)

[3.6.2 Routine screening under 5-yearly interval (Primary HPV screening) 40](#_Toc448766821)

[3.7 Screening, diagnosis and treatment cost 41](#_Toc448766822)

[3.8 QALY weights 43](#_Toc448766823)

[3.8.1 QALY weights set 1 43](#_Toc448766824)

[3.8.2 QALY weights set 2 44](#_Toc448766825)

[3.8.3 QALY weights set 3 45](#_Toc448766826)

[3.9 Vaccination coverage 46](#_Toc448766827)

[4 MODEL CALIBRATION AND VALIDATION 47](#_Toc448766828)

[4.1 Calibration of natural history model without screening 47](#_Toc448766829)

[4.1.1 HPV prevalence 47](#_Toc448766830)

[4.1.2 Cervical cancer incidence in unscreened population 48](#_Toc448766831)

[4.2 Calibration of the screening model 49](#_Toc448766832)

[4.2.1 Cervical cancer incidence and mortality 50](#_Toc448766833)

[4.2.2 Cervical cancer stage distribution 51](#_Toc448766834)

[4.2.3 HPV type distribution in cervical cancer 51](#_Toc448766835)

[4.2.4 Histologically-confirmed abnormalities 52](#_Toc448766836)

[4.2.5 HPV type distribution among women diagnosed with high-grade lesion 53](#_Toc448766837)

[4.2.6 Comparing other model’s predictions with data observed in NZ 54](#_Toc448766838)

[4.3 Calibration of cytology test performance assumption 55](#_Toc448766839)

[4.3.1 Test yield 55](#_Toc448766840)

[4.3.2 Positive predictive value of high-grade cytology 56](#_Toc448766841)

[4.4 Calibration of screening participation assumption 56](#_Toc448766842)

[5 SUPPLEMENTARY ANALYSIS 57](#_Toc448766843)

[6 SENSITIVITY ANALYSIS 63](#_Toc448766845)

[6.1 Strategy S2a, no vaccination 64](#_Toc448766846)

[6.2 Strategy S2c, no vaccination 68](#_Toc448766847)

[6.3 Strategy S2a, vaccination 71](#_Toc448766848)

[6.4 Strategy S2c, vaccination 74](#_Toc448766849)

# OVERVIEW OF MODEL PLATFORM

The model platform used in this evaluation contains several elements, including:

1. A dynamic model of sexual behaviour, HPV transmission and HPV vaccination in females and males in the New Zealand population.
2. A model of the natural history of cervical HPV infection, progression, regression, the development of cervical intraepithelial neoplasia (CIN) and invasive cervical cancer.
3. A model of cervical screening, management, diagnosis and treatment of CIN, and ‘test-of-cure’ after treatment of high grade CIN; and
4. A population component that applies demographic data to the outputs to estimate cross-sectional results in each age group after the implementation of each potential new screening strategy.

The model simulated a cohort of women from age 10 to 89 years at annual time step. The whole cohort was assumed to be ‘perfectly healthy’ (i.e. no CIN or HPV infection.) at 10 year-old when the simulation began. Each year after the simulation started, a proportion of the ‘perfectly healthy’ cohort became infected with HPV according to the HPV incidence rate estimated by the dynamic HPV transmission model, which simulates the transmission of HPV infection and the effect of HPV vaccination in the New Zealand population. In the model, most HPV infections were regressed spontaneously but some were progressed to develop CIN and cervical cancer. The modelled HPV and CIN natural history is depicted in Figure 1. Detailed information of the modelled HPV and CIN natural history are provided in page 4.

Cervical screening begins from age 20 or 25 years in the model depends on the strategy evaluated. Women who participated in screening were screened by cytology and/or HPV screening test depends on the strategy evaluated. Based on the women underlying health state and the characteristics of the screening test(s) (see page 26-37 for more information related to the screening and diagnostics tests accuracy assumptions), women with negative screening test outcome were returned to routine screening; women with positive test outcome were either referred to further colonoscopy examination (and biopsy if needed) or follow-up with another cytology and/or HPV test in 12 months depends on the strategy evaluated. Women who attended colposcopy and had a negative or CIN1 outcome were follow-up in 12 months; women diagnosed with CIN2/3 were treated for the precancer lesions and follow-up in 12 months under ‘test-of-cure’ management; women diagnosed with cervical cancer were referred to cancer treatment. Detailed information related to the modelled screening strategies and follow-up managements are provided in *Section 2 Strategies Flow Chart*. The model incorporates local data on screening participation and follow-up management compliance based on the analyses of the National Cervical Screening Program Registry data in 2012 (see page 39-41 for more information).

The model platform has previously gone through multiple rounds of update, calibration and validation during the past and was used to evaluate changes to the cervical screening interval in Australia and United Kingdom,[1,2] to assess the role of alternative technologies for primary screening in Australia and England, [1,3,4] to assess the role of HPV triage testing for women with low-grade cytology in Australia and New Zealand, [5] to estimate the overall cervical screening program cost in Australia, [6] to access the cost-effectiveness of primary HPV screening in England and Australia, [3,4] and to evaluate the cost-effectiveness of alternative screening strategies and combined screening and vaccination approaches in rural China. [7,8]. . Detailed model parameter assumptions are provided in the later section of this appendix.

Figure 1 Schematic diagram for model structure


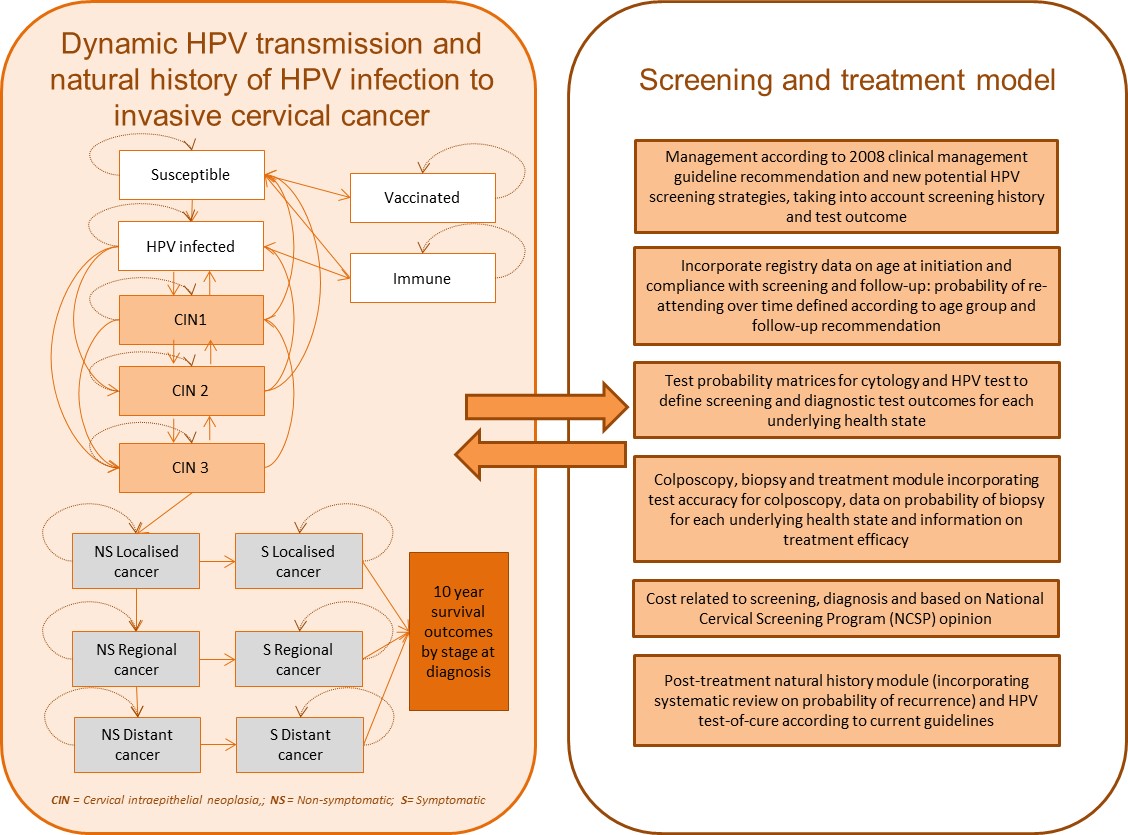


Figure above was adapted from Figure 1 of Creighton et al 2010[9] and modified to reflect the structure of the model for this evaluation.

# STRATEGIES FLOW CHARTS

## Current practice

The comparator for the evaluation is the current cervical screening program in NZ (NZ). The current practice of National Cervical Screening Program (NCSP) recommends 3-yearly screening with cytology (with ASC-US/LSIL HPV triage for women aged 30 years or above) for 20-69 years sexually active women [10]. A model of current NCSP was constructed based on the latest clinical management guideline recommendation [10] (see Figure 2).

Figure 2 Screening management for current practice (primary screening with cytology test for 20-69 years at 3-yearly interval)

## Primary HPV testing strategies

A total of 16 primary HPV screening strategies, determined in a series of consultations with the NCSP and its Advisory Group were modelled. The strategies can be grouped into four main groups. The first group (S1) assumed primary HPV screening with cytology triage for all high-risk (HR) HPV positive samples. The second group (S2) assumed primary HPV screening (with partial genotyping) with cytology triage for non-16/18 HR HPV positive samples. The third group (S3) assumed primary screening with HPV and cytology co-testing and the fourth group (S4) assume HPV (with partial genotyping) and cytology co-testing.

Each strategy group contained four sub-strategies representing a combination of assumptions for screening start age (20 or 25 years), primary screening strategy (primary HPV screening for all ages or cytology screening for cytology screening or 20-29 year-olds switching to HPV screening in older women, i.e. ‘switchover strategies’), and management for intermediate risk group (follow-up with HPV and cytology co-testing in 12 months or refer to colposcopy immediately).

The detailed screening management of the 16 primary HPV screening strategies modelled in this evaluation are provided in Figure 3 to Figure 18.

Figure 3 Screening management for strategy S1a (primary screening with HPV testing alone for 25-69 years, 12 months follow-up option)

Figure 4 Screening management for strategy S1b (primary screening with HPV testing alone for 25-69 years, immediate colposcopy option)

Figure 5 Screening management for strategy S1c (cytology screening for 20-29 years, primary screening with HPV testing alone for 30-69 years, 12 months follow-up option)

Figure 6 Screening management for strategy S1d (cytology screening for 20-29 years, primary screening with HPV testing alone for 30-69 years, immediate colposcopy option)

Figure 7 Screening management of S2a (primary screening with HPV testing and partial genotyping for 25-69 years, 12 months follow-up option)

Figure 8 Screening management of strategy S2b (primary screening with HPV testing and partial genotyping for 25-69 years, immediate colposcopy option)

Figure 9 Screening management of strategy S2c (cytology screening for 20-29 years, primary HPV screening with partial genotyping for 30-69 years, 12 months follow-up option)

Figure 10 Screening management of strategy 2d (cytology screening for 20-29 years, primary HPV screening with partial genotyping for 30-69 years, immediate colposcopy option)

Figure 11 Screening management of strategy 3a (primary screening with HPV and cytology co-testing for 25-69 years, 12 months follow-up option)

Figure 12 Screening management of strategy S3b (primary screening with HPV and cytology co-testing for 25-69 years, immediate colposcopy option)

Figure 13 Screening management of strategy 3c (cytology screening for 20-29 year, primary screening with HPV and cytology co-testing for 30-69 years, 12 months follow-up option)

Figure 14 Screening management of strategy 3d (cytology screening for 20-29 years, primary screening with HPV and cytology co-testing for 30-69 years, immediate colposcopy option)

Figure 15 Screening management of strategy 4a (primary screening with HPV with partial genotyping and cytology co-testing and for 25-69 years, 12 months follow-up option)

Figure 16 Screening management of strategy 4b (primary screening with HPV with partial genotyping and cytology co-testing for 25-69 years, immediate colposcopy option)

Figure 17 Screening management of strategy 4c (cytology screening for 20-29 years, primary screening with HPV with partial genotyping and cytology co-testing for 30-69 years, 12 months follow-up option)

Figure 18 Screening management of strategy 4d (cytology screening for 20-29 years, primary screening with HPV with partial genotyping and cytology co-testing for 30-69 years, immediate colposcopy option)

## Follow-up in women with abnormal screening outcome

The modelled follow-up managements for women with abnormal screening outcome are described in Figure 19 to Figure 25. The management under current cervical screening program was modelled based on the latest NZ clinical management guideline recommendation [10]. NCSP opinion was sought for the managements that are not specified in the guideline.

Figure 19 Colposcopy management for women referred with non-HG cytology test result (for current practice)

Figure 20 Colposcopy management for women referred with HG cytology test result (for current practice)

Figure 21 Colposcopy management for women referred with non-HG cytology test result (for primary HPV screening strategies)

Figure 22 Colposcopy management for women referred with high-grade cytology test result (for primary HPV screening strategies)

Figure 23 Follow-up management for 3 months repeat cytology and colposcopy test management for women whose last screening outcome was HG discordant

Figure 24 Follow-up management for 12 months repeat cytology test & colposcopy for women whose last cytology screening outcome was not high-grade and whose colposcopy findings was unsatisfactory/satisfactory and normal or whose histology outcome was not high-grade

Figure 25 Follow-up management for 12 months repeat HPV and cytology test for women whose last screening outcome was associated with normal colposcopy findings or normal/low-grade histology (for primary HPV screening strategies)

## Follow-up management for women treated for high-grade CIN

The modelled follow-up managements for women treated for high-grade CIN are described in Figure 26 to Figure 31. The management under current cervical screening program was modelled based on the NZ clinical management guideline recommendation [10]. NCSP opinion was sought for the managements that are not specified in the guideline.

Figure 26 Post-treatment test-of-cure follow-up management for women treated for HG CIN – 12 months post-treatment

Figure 27 Post-treatment test-of-cure follow-up management for women treated for HG CIN – 24+ months post-treatment and who previously tested negative for both HPV and cytology

Figure 28 Post-treatment test-of-cure follow-up management for women treated for HG CIN – 24+ months post-treatment and who previously tested negative for HPV infection but ASC-US/LSIL cytology

Figure 29 Colposcopy management for women under post-treatment test-of-cure management and referred with non-HG cytology test result

Figure 30 Colposcopy management for women under post-treatment test-of-cure management and referred with HG cytology test result

Figure 31 Follow-up management for 3 months repeat cytology and colposcopy test for women under post-treatment test-of-cure management with HG discordant result

# DATA SOURCES

## HPV incidence and CIN natural history model

The model used in this evaluation was based on the model used in a previous assessment performed for the NZ Ministry of Health [11]. The type-specific model contains three separate natural history models for oncogenic HPV types to which the vaccines apply, namely, (i) *HPV 16*, (ii) *HPV 18* and (iii) Other high risk HPV types (*HPV OHR*); vaccine provides protection against HPV 16 and 18 infections. This multi-type natural histories take into account of three different HPV genotype groupings, representing infection with HPV16 (with or without a co-infection of other oncogenic HPV type (including HPV 18)), infection with HPV 18 (with or without a co-infection of other oncogenic HPV type but not type 16), and infection with one or more high-risk HPV types other than HPV 16 or HPV 18, respectively. Detailed model calibration and validation outcome are provided in the later section of this appendix.

## Cancer survival

We modelled cervical cancer by the extent of disease. The stage-specific survival of cancer patients was modelled based on data observed in NZ in the period from 1994-2003 [12] and adjusted to fit to the cervical cancer mortality observed in NZ.

## Mortality and hysterectomy rate

The age-specific hysterectomy rates were derived from Paul et al. [13] who modelled the hysterectomy fraction by age over time using data from hysterectomy procedures in private and public hospitals in NZ. The age-specific rate of deaths from causes other than cervical cancer was calculated from the all-cause mortality rate after subtracting the cervical cancer mortality rate. [14]

## Screening and diagnostic test characteristics and data sources

### Liquid-based cytology (LBC) test

The base case value assumed for unsatisfactory rate of LBC was 1.17% based on the average cytology unsatisfactory rate observed between January 2010 and June 2013 in NZ [15,16,17,18,19,20,21]. A range of 0.6-2% investigated in sensitivity analysis was obtained using the lowest and highest rate observed during the same time period in NZ.

We use a test probability matrix (TPM) to model LBC test characteristics, which relates a probability of getting a negative, ASC-US, LSIL, ASC-H and HSIL outcome depending on women’s modelled true underlying health state (i.e. normal, infected with HPV but no CIN, CIN1, CIN2, CIN3 and cancer). This modelling thus mirrors differences in the mix of underlying health states seen when a group of women are having the test for primary screening purposes or, alternatively, for triage testing after having a HPV positive result. The modelled TPM of LBC was derived by calibrating to the cytology abnormality rate (Table 1 and Figure 31) as well as the positive predictive value of high-grade cytology observed in 2010-11 [22], accounting for the effect of current cervical screening in NZ. The modelled HPV triage test positivity rate on ASC-US and LSIL tests were also consistent with data observed in NCSP in 2012-13 (see Figure 36). [19,20,21,23]

Table 1 Estimated test yields for base case liquid-based cytology test characteristics assumption in satisfactory cytology tests from women aged 20-69 years, compared to observed data in NZ in 2010-11

| **Estimated outcome versus observed data** | **Cytology outcome** | | | | |
| --- | --- | --- | --- | --- | --- |
|  | **Negative** | **ASC-US** | **LSIL** | **ASC-H** | **HSIL** |
| Estimated outcome | 92.3% | 2.4% | 3.8% | 0.5% | 0.9% |
| *Observed data 2010 [22]* | *92.7%* | *2.4%* | *3.6%* | *0.5%* | *0.8%* |
| *Observed data 2011[22]* | *92.9%* | *2.2%* | *3.6%* | *0.5%* | *0.8%* |

Figure 32 Estimated age-specific rate of (a) ASCUS, (b) LSIL, (c) ASC-H and (d) HSIL among women screened, compared to observed data in NZ in 2010-11

|  |  |
| --- | --- |
|  |  |

Table 2 Estimated positive predictive value for ASC-H or worse of base case liquid-based cytology test characteristics assumption in satisfactory cytology tests from women aged 20-69 years, compared to observed data in NZ in 2011

| **Estimated outcome versus observed data** | **Cytology outcome** | |
| --- | --- | --- |
|  | **ASC-H or worse** | **HSIL or worse** |
| Estimated outcome | 70.6% | 83.0% |
| *Observed data 2010 [22]* | *70.4%* | *82.4%* |
| *Observed data 2011[22]* | *70.6%* | *82.9%* |

Table 3 shows the sensitivity and specificity derived from the modelled TPM of LBC at different cytological thresholds for detecting a CIN2 or worse (CIN2+) and CIN3 or worse (CIN3+) health state when used for primary screening and triage testing for women with positive HPV test outcome. When LBC was used for primary screening, at cytological threshold of ASC-US, the derived test sensitivity and specificity for detecting CIN2+ are 78.6% and 94.3%, respectively and the corresponding rates for detecting CIN3+ are 79.0% and 93.8%, respectively in women age 20-69 years, assuming colposcopy and histology verification has perfect accuracy. The modelled LBC was associated with similar test sensitivity but lower test specificity when it was used as a triage test, compared to the LBC test for primary screening.

Table 3 Modelled sensitivity and specificity of liquid-based cytology in women age 20-69 years (in the base case – sensitivity analysis used to assess the impact of alternative assumptions)

| **Test Threshold** | **CIN2+** | | **CIN3+** | |
| --- | --- | --- | --- | --- |
|  | **Sensitivity^*^** | **Specificity^*^** | **Sensitivity^*^** | **Specificity^*^** |
| *For primary screening* | | | | |
| ASC-US | 78.6% | 94.3% | 79.0% | 93.8% |
| LSIL | 73.3% | 96.5% | 77.0% | 96.1% |
| ASC-H | 47.5% | 99.6% | 49.0% | 99.3% |
| *For triage all high-risk HPV positive* | | | | |
| ASC-US | 78.7% | 74.5% | 79.0% | 72.1% |
| LSIL | 74.0% | 81.2% | 77.0% | 79.1% |
| ASC-H | 47.8% | 97.7% | 49.0% | 95.8% |

*^*^ The test sensitivities and specificities were calculated by multiplying the test probability matrix with the underlying health state distribution of the women targeted for the test, assuming colposcopy and histology verification has perfect accuracy*

The woman’s true underlying health state changes overtime due to the dynamic in HPV infection/clearance and CIN progression/regression. The test sensitivity and specificity therefore also vary by age due to the changes in the true underlying health state among women in that age group. Figure 33 and Figure 34 show the modelled age-specific test specificity and sensitivity of LBC for primary screening and triage testing for women with positive HPV result. The test specificity of LBC is lower in women aged <30 years and increased in women aged >= 30 years, the test sensitivity remains similar across all ages.

Figure 33 Modelled age-specific (a) test specificity and (b) sensitivity of LBC for primary screening in by different cytology and histology cut-off^*^

| 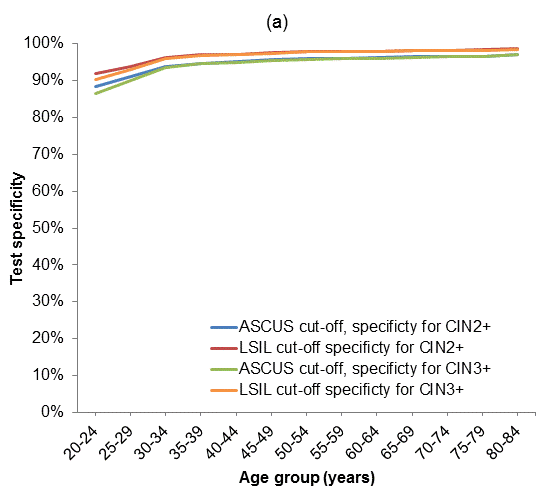 | 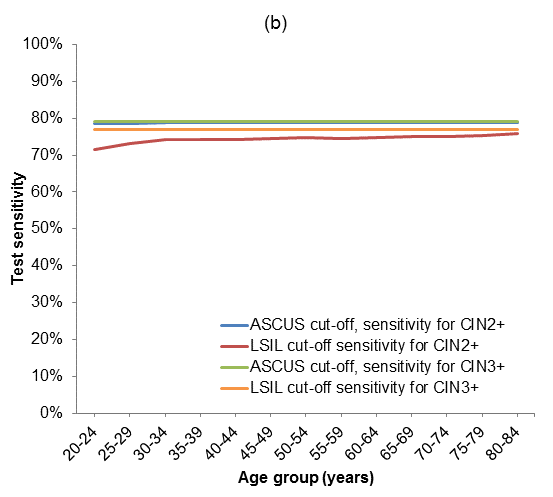 |
| --- | --- |

*^*^ The test sensitivities and specificities were calculated by multiplying the test probability matrix with the underlying health state distribution of the women targeted for the test, assuming colposcopy and histology verification has perfect accuracy*

Figure 34 Modelled age-specific (a) test specificity and (b) sensitivity of LBC for triage testing for women with positive HPV result in by different cytology and histology cut-off^*^

| 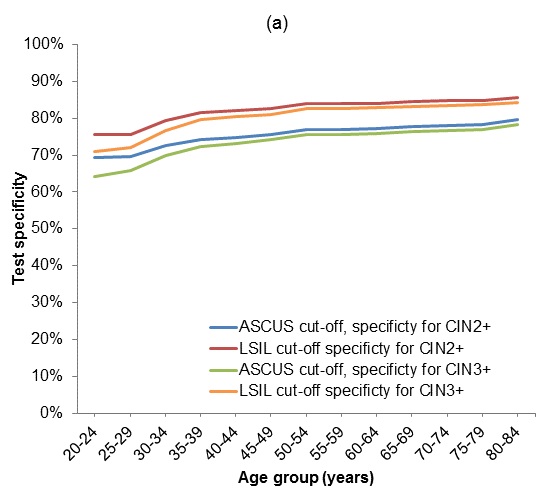 | 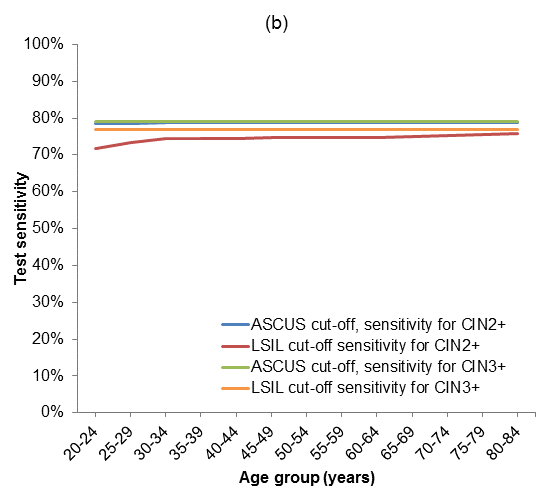 |
| --- | --- |

*^*^ The test sensitivities and specificities were calculated by multiplying the test probability matrix with the underlying health state distribution of the women targeted for the test, assuming colposcopy and histology verification has perfect accuracy*

An alternative test probability matrix of LBC assuming higher test sensitivity in detecting CIN2+ was derived for the sensitivity analysis to assess the impact of increased test sensitivity of triage LBC testing women with positive HPV test outcome on the study’s findings (Table 2).

Table 4 Modelled sensitivity and specificity of best case liquid-based cytology for triage women with positive HPV test outcome, in age 20-69 years

| **Test Threshold** | **CIN2+** | | **CIN3+** | |
| --- | --- | --- | --- | --- |
|  | **Sensitivity^*^** | **Specificity^*^** | **Sensitivity^*^** | **Specificity^*^** |
| ASC-US | 84.0% | 74.5% | 84.0% | 72.9% |
| LSIL | 79.3% | 81.2% | 82.0% | 78.8% |
| ASC-H | 51.3% | 97.7% | 54.0% | 95.8% |

*^*^ The test sensitivities and specificities were calculated by multiplying the test probability matrix with the underlying health state distribution of the women targeted for the test, assuming colposcopy and histology verification has perfect accuracy*

### HPV test for primary screening

The modelled base case primary HPV test has a sensitivity of 96.4% and a specificity of 90.3% for CIN2+ detection, and 98.4% and 89.7%, respectively for CIN3+ detection (Table 5). This test accuracy assumption was calibrated to the pooled absolute test sensitivity and specificity reported in a meta-analysis published in 2012 [24].

The modelled primary HPV test was more sensitive but less specific in detecting CIN 2+ and CIN 3+ compared to the base case LBC (Table 5). Compared to the base case LBC test characteristics assumption at ASCUS+ threshold, the modelled HPV test has a relative sensitivity of 1.23 and a relative specificity of 0.96 for CIN2+ detection. The corresponding rates were 1.25 and 0.96, respectively for CIN3+ detection (Table 5). This modelled relative test performance is consistent with the lower end of the meta-analysis findings [24] (Table 5), and is a favourable assumption strategy assuming cytology screening (i.e. current practice) in this evaluation.

A best case primary HPV test, which assumed to have a test characteristics consistent with the upper ends of the meta-analysis findings[24] on both the absolute and the relative (to cytology) test sensitivity and specificity of HPV test for primary screening was investigated in the sensitivity analysis. A worst case primary HPV test characteristic based on the same test accuracy assumed for the base case triage HPV test for low-grade cytology was also include for sensitivity analysis. The different test sensitivity and specificity derived from the worst case primary HPV test and base case triage HPV test were due to the differences in the mix of underlying health states among the women targeted for the test.

Table 5 Modelled test characteristics of HPV test for primary screening in women aged 20-69 years

| **Test characteristics** | **Base case** | | **Worse case** | | **Best case** | | ***Data observed***  ***(95% CI)*** *)[24]* | |
| --- | --- | --- | --- | --- | --- | --- | --- | --- |
|  | **Sens.^*^** | **Spec. ^*^** | **Sens. ^*^** | **Spec. ^*^** | **Sens. ^*^** | **Spec. ^*^** | ***Sens.*** | ***Spec.*** |
| *For CIN2+ detection* | | | | | | | | |
| Absolute | 96.4% | 90.3% | 93.4% | 90.3% | 98.6% | 92.6% | *96%*  *(95-98%)* | *91%*  *(90-93%)* |
| Relative to LBC (ASCUS+) | 1.23 | 0.96 | 1.19 | 0.96 | 1.25 | 0.98 | *1.37*  *(1.22-1.54)* | *0.97*  *(0.96-0.98)* |
| Relative to LBC (LSIL+ ) | 1.31 | 0.94 | 1.27 | 0.94 | 1.35 | 0.96 | *1.40*  *(1.27-1.54)* | *0.92*  *(0.90-0.94)* |
| *For CIN3+ detection* | | | | | | | | |
| Absolute | 98.4% | 89.7% | 95.9% | 89.7% | 99.0% | 92.0% | *98%*  *(97-99%)* | *N/A*^†^ |
| Relative to LBC (ASCUS+) | 1.25 | 0.96 | 1.21 | 0.96 | 1.25 | 0.98 | *1.43*  *(1.15-1.77)* | *0.97*  *(0.96-0.99)* |
| Relative to LBC (LSIL+ ) | 1.28 | 0.93 | 1.25 | 0.93 | 1.29 | 0.96 | *1.36*  *(1.21-1.53)* | *0.97*  *(0.96-0.98)* |

ASCUS+ - cytologically threshold of ASCUS or worse; LSIL+ - cytologically threshold of LSIL or worse; Sens –sensitivity; Spec-specificity

*† Data not available in the meta-analysis*

*^*^ The test sensitivities and specificities were derived by multiplying the test probability matrix with the underlying health state distribution of the women targeted for the test, assuming colposcopy and histology verification has perfect accuracy*

Figure 35 shows the modelled age-specific test specificity and sensitivity of HPV test for primary HPV screening. The test specificity was lowest in 20-24 year-old (76-78%) and increased by age. The test sensitivity was similar across ages.

Figure 35 Modelled age-specific (a) test specificity and (b) sensitivity of HPV test for primary screening at different histology cut-off^*^

| 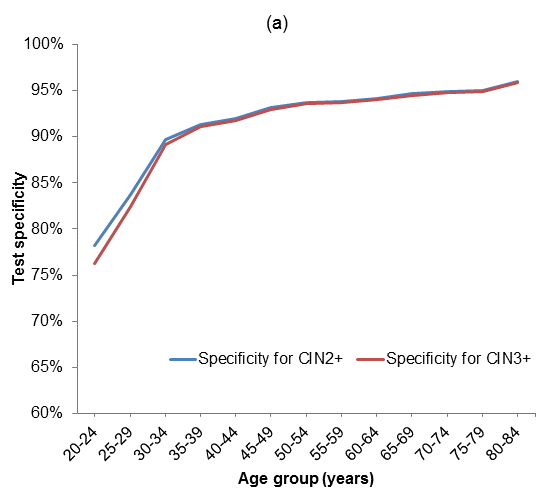 | 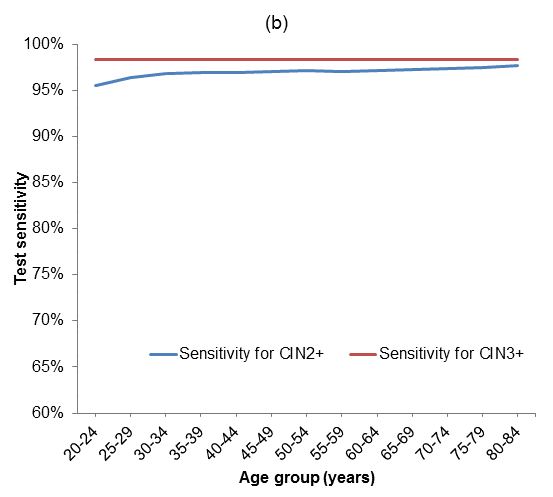 |
| --- | --- |

*^*^ The test sensitivities and specificities were derived by multiplying the test probability matrix with the underlying health state distribution of the women targeted for the test, assuming colposcopy and histology verification has perfect accuracy*

### Triage HPV test for low-grade cytology

The modelled base case triage HPV test for low-grade cytology were calibrated to the observed age-specific HPV positive rate among women with ASCUS or LSIL cytology result in NZ [19,20,21,23] and the test sensitivity findings of Arbyn et al.[24]. Same test accuracy was modelled for HPV triage testing for both women with ASC-US and LSIL cytology result. Differences in the test sensitivity and specificity were due to the differences in the mix of underlying health states between women with ASC-US and LSIL cytology result. Figure 36 below compares the calibrated model’s prediction and data observed in NZ in the period between 2012 and 2013.[19,20,21,23]

Figure 36 Predicted percentage of HPV positive among (a) cytology ASCUS and (b) cytology LSIL samples, compared with data observed in NZ

|  |  |
| --- | --- |
|  | |

The calibrated base case HPV test for cytology ASCUS has a test sensitivity of 90.8% and a specificity of 72.6% for CIN2+ detection. The corresponding rates were 95.9% and 71.0%, respectively for CIN3+ detection (see Table 6). The modelled test sensitivity and specificity of HPV test for LSIL were 94.1% and 47.4%, respectively for CIN2+ detection and 95.9% and 46.1%, respectively for CIN3+ detection. The test sensitivity of triage HPV test for both ASCUS and LSIL cytology are consistent with the findings of Arbyn et al. [24]. However, as a result of calibration, the modelled triage HPV tests for both ASCUS and LSIL assumed a higher test specificity compared to the meta-analysis’s findings [24] (see Table 6).

A worse case triage HPV test assumed a lower test sensitivity and specificity was derived for the sensitivity analysis (see Table 6). It was derived based on the lower end of the 95% CI associated with pooled test sensitivity and specificity findings of the meta-analysis [24]. A best case triage HPV assumed the same test accuracy as the modelled base case HPV test for primary screening was also include for sensitivity analysis. The different test sensitivity and specificity derived from the best case triage HPV test and the base case primary HPV test were due to the differences in the mix of underlying health states among the women targeted for the test.

Table 6 Modelled test characteristics of triage HPV test for cytology ASCUS and LSIL

| **Test characteristics** | **Base case** | | **Worse case** | | **Best case** | | ***Data observed***  ***(95% CI)****[24]* | |
| --- | --- | --- | --- | --- | --- | --- | --- | --- |
|  | **Sens. ^a,b^** | **Spec. ^a,b^** | **Sens. ^a,b^** | **Spec. ^a,b^** | **Sens. ^a,b^** | **Specs. ^a,b^** | **Spec. ^a,b^** | ***Spec.*** |
| *For CIN2+ detection* | | | | | | | | |
| Triage ASCUS | 90.8% | 72.6% | 89.5% | 50.6% | *94.4%* | *72.6%* | *90.4% (88.1- 92.3%)* | *58.3% (53.6-62.9%)* |
| Triage LSIL | 94.1% | 47.4% | 90.5% | 24.6% | *97.0%* | *47.4%* | *95.4% (94- 96.5%)* | *27.8% (23.8-32.1%)* |
| *For CN3+ detection* | | | | | | | | |
| Triage ASCUS | 95.9% | 71.0% | 91.0% | 49.6% | *98.4%* | *70.9%* | *93.7% (90.4- 95.9%)* | *52.3% (45.7-58.7%)* |
| Triage LSIL | 95.9% | 46.1% | 91.0% | 24.2% | *98.4%* | *46.0%* | *96.4% (90.5- 98.7%)* | *23.7% (19.4-28.7%)* |

ASCUS- cytology ASCUS; LSIL- cytology LSIL; Sens- Sensitivity; Specs- specificity;

*^a^ Same test accuracy was modelled for HPV triage testing for both women with ASC-US and LSIL cytology result. Differences in the test sensitivity and specificity were due to the differences in the mix of underlying health states between women with ASC-US and LSIL cytology result.*

*^b^ The test sensitivities and specificities were derived by multiplying the test probability matrix with the underlying health state distribution of the women targeted for the test, assuming colposcopy and histology verification has perfect accuracy*

Figure 37 shows the modelled age-specific test specificity and sensitivity of HPV triage testing for women with ASC-US or LSIL cytology result. The modelled test specificity was estimated to increase by age; test sensitivity was found to remain stable across ages. Thse model predictions are consistent with the findings of an international meta-analysis conducted by Arbyn et al.[25], which found the test specificity always increased by age, based on the results of three studies[26,27,28], but that the test sensitivity of triage HPV test did not vary significantly by age group. The model predictions were also consistent with the increased triage HPV test specificity by age group found in the ATHENA trial[29]; and the similar sensitivity for both CIN2+ and CIN3+ detection across age groups in women aged 21-39 years.  While the authors reported an observed decrease in test sensitivity for both CIN2+ and CIN3+ detection in women aged >= 40+ years, they explained that this was “likely owing to pathologic misclassification of CIN 2 or worse in older women” [29]

Figure 37 Modelled age-specific (a) test specificity and (b) sensitivity of HPV testing for women with ASC-US or LSIL cytology result by different histology cut-off^*^

| 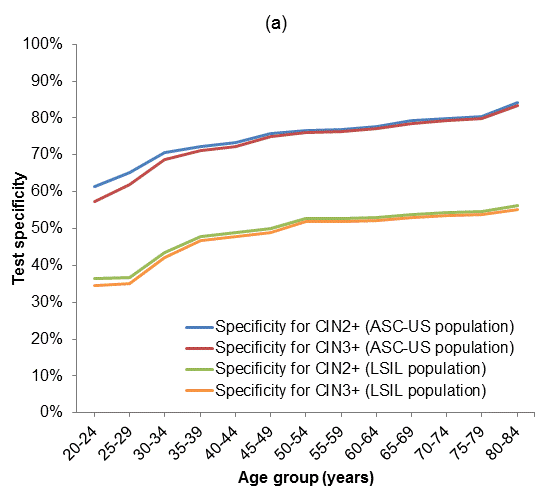 | 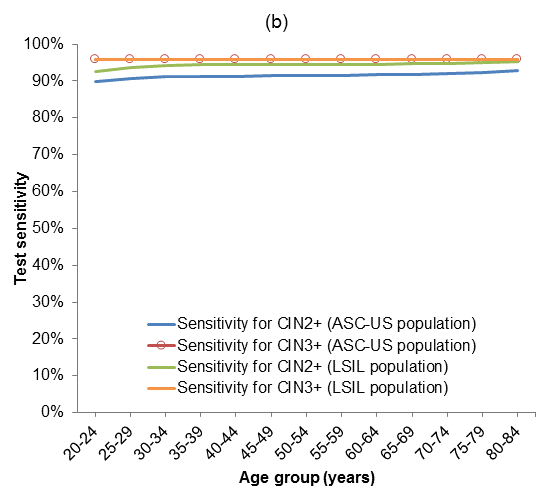 |
| --- | --- |

*^*^ The test sensitivities and specificities were derived by multiplying the test probability matrix with the underlying health state distribution of the women targeted for the test, assuming colposcopy and histology verification has perfect accuracy*

### HPV test for follow-up women treated for high-grade lesion

The modelled HPV test for follow-up women treated for high-grade lesions was also derived based on the findings of Arbyn et al [24]. The base case HPV test has a sensitivity of 92.5% and a specificity of 82.7% for CIN2+detection, and 93.1% and 82.2%, respectively for CIN3+ detection (Table 7). Alternative sets of test characteristics were also derived for sensitivity analysis, based on the 95% CI reported in the meta-analysis [24].

Table 7 Modelled test characteristics of HPV test for follow-up women treated for high-grade lesion

| **Test characteristics** | **Base case^*^** | | **Worse case^*^** | | **Best case^*^** | | ***Data observed***  ***(95% CI)****[24]* | |
| --- | --- | --- | --- | --- | --- | --- | --- | --- |
|  | **Sens.** | **Spec.** | **Sens.** | **Spec.** | **Sens.** | **Spec.** | ***Sens*** | ***Spec.*** |
| For CIN2+ detection | 92.5% | 82.7% | 85.1% | 75.3% | 96.7% | 86.2% | *92.9% (85-96.8%)* | *81.3% (74.7-86.5%)* |
| For CIN3+ detection | 93.1% | 82.2% | 86.2% | 75.0% | 98.0% | 85.7% | *N/A^†^* | *N/A^†^* |

Sens- Sensitivity; Specs- specificity;

*† Data not available in the meta-analysis*

*^*^ The test sensitivities and specificities were derived by multiplying the test probability matrix with the underlying health state distribution of the women targeted for the test, assuming colposcopy and histology verification has perfect accuracy*

### Colposcopy

A test probability matrix for colposcopy was derived specifying the relationship between each possible underlying natural history health state at the time of testing and the probability of colposcopy result being abnormal. The baseline estimates were obtained from a large colposcopy dataset (over 21,000 colposcopies) supplied by the Royal Women’s Hospital in Victoria [5,30]. An alternative set of test accuracy assumption with higher rate of having abnormal result at colposcopy evaluation were derived based on the findings of the HPV Sentinel sites study in the UK [31] for sensitivity analysis (Table 8). Another set of test accuracy assumption assuming colposcopy test positive rate is 10% lower than the base case assumption was also investigated.

Table 8 Modelled test characteristic of colposcopy

| **Women underlying health state** | **Probability of having abnormal result at colposcopy evaluation** | |
| --- | --- | --- |
|  | **Base case (%)** | **Range for sensitivity analysis (%)** |
| Normal | 50.2 | 45.2-73.8 |
| HPV | 50.2 | 45.2-73.8 |
| CIN1 | 76.5 | 68.9-79.2 |
| CIN 2+ | 88.4 | 79.6-90.8 |

The model incorporated age-specific estimates of the proportion of colposcopies with no or partial visualisation of the transformation zone, based on an analysis of ~54,000 colposcopies recorded on the NCSP Register which occurred in NZ during 2011-2012 (see Table 9).

Table 9 Modelled age-specific probability of colposcopy being unsatisfactory

| **Age group (years)** | **Probability of colposcopy being unsatisfactory (%)** |
| --- | --- |
| 15-19 | 5% |
| 20-24 | 5% |
| 25-29 | 7% |
| 30-34 | 10% |
| 35-39 | 12% |
| 40-44 | 17% |
| 45-49 | 23% |
| 50-54 | 32% |
| 55-59 | 44% |
| 60-64 | 52% |
| 65-69 | 56% |
| 70+ | 66% |

## Screening initiation assumptions

The modelled age-specific screening initiation in women aged younger than 25 years was obtained by analysing of the NCSP-R data in 2012. The analysis found a small proportion (~5%) of women in NZ started cervical screening before age 20 years. Among these women who started screening earlier than the recommended age, majority of them (>80%) had their first screening test at 18-19 years. The screening initiation assumption in women aged 26 or older were obtained via calibrating the model predictions of 3- and 5-years screening participation rate observed in 2012 in NZ (see section 4.4 for calibration outcome). This screening initiation assumption was used for baseline model calibration and validation.

In the base case analysis, the strategies that modelled screening starts from age 20 years, including current practice strategy and the ‘switch-over’ strategies, was assumed with a similar screening initiation assumption but without early screening initiation before 20 years-old. In these strategies, we assumed the group of women who started screening before age 20 years would have their first screening test at 20 years-old.

For the strategies that assumed screening starts at 25 years, we modelled a ‘rapid uptake’ at 25 years by assuming women who currently had started screening before age 25 years would all have their first screening test at 25 year-old in the base case analysis. A ‘gradual uptake’ assumption, which assumed a ‘slower’ screening initiation rate at 25-29 years, was explored in the sensitivity analysis. The proportion of women ever-screened was assumed to remain the same as per current practice in both of the ‘rapid uptake’ and ‘gradual uptake’ assumptions.

The modelled age-specific screening initiation rate and cumulative proportion of women who have attended screening at least once (‘ever-screened’) for current practice and primary HPV screening strategies are shown in Figure 38.

Figure 38 Modelled (a) age at initiating screening and (b) proportion of women ever screened, by age.

|  |
| --- |
|  |

## Screening compliance assumptions

### Routine screening at 3-yearly interval (Current practice)

We used data from the NCSP register to determine the proportion of women who re-attend for a smear given their last test result. For women under routine screening, we calculated the annual probability that women return for their next smear after their last negative routine smear. More details of the methods can be found in the 2012 Annual Report for the National Cervical Screening Programme [32]. Using these rates of return, the model predicts 3- and 5-year coverage rates that compare very well with observed coverage rates in NZ as shown in Figure 39 and Figure 40.

Figure 39: Observed and predicted 3-year screening coverage rates in NZ


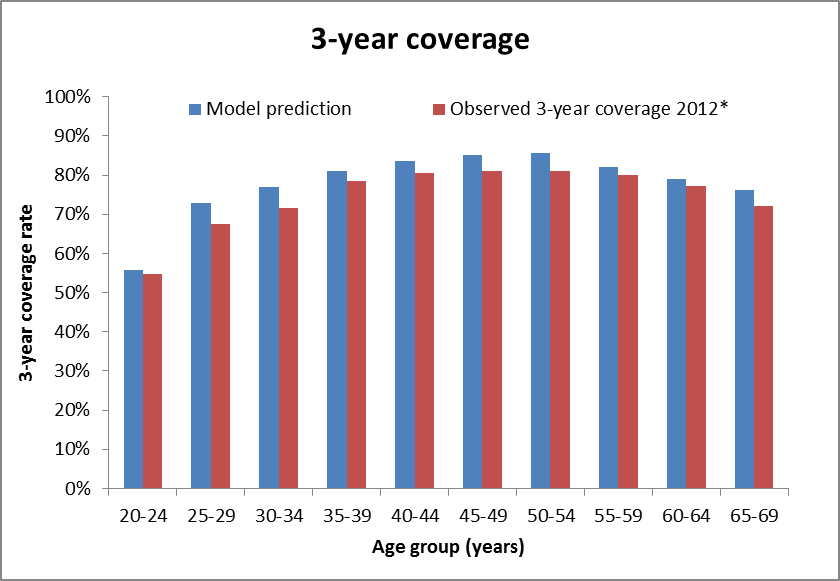

*Coverage data taken from observed rates in NZ [19]

Figure 40: Observed and predicted 5-year screening coverage rates in NZ


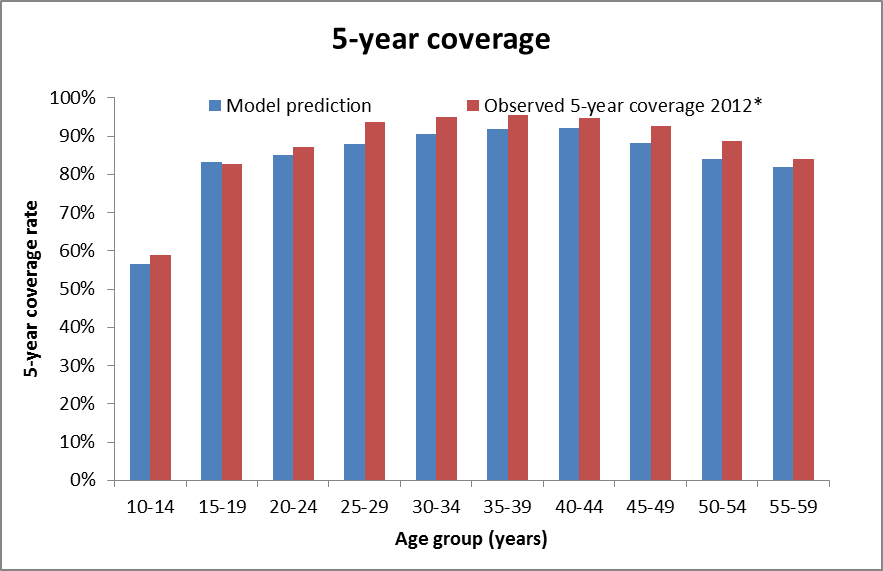

*Coverage data taken from observed rates in NZ [19]

### Routine screening under 5-yearly interval (Primary HPV screening)

We assume a reminder-based program for 5-yearly screening in NZ. We assume that the cumulative proportion of women who attend 1 year early under primary HPV screening at 5-yearly intervals (i.e., who attend 4 years after their last normal routine screening test) is equivalent to the proportion who have attended within two years of their last normal routine screening test under current practice management in NZ. We assume an equivalent proportion turn up ‘on-time’ and one-year late as observed under current practice management in NZ. A scenario with more on-time screening and less early re-screening (‘very good compliance’) was also explored in sensitivity analysis. In this scenario, the number of women who attend early or on-time was based on the screening behaviour pattern observed in England (methods described previously [4]). The cumulative re-screening probabilities under current practice management and 5-yearly HPV screening is shown in Figure 41.

Figure 41: Cumulative re-screening probabilities assumed for primary HPV screening at 5-yearly intervals in NZ


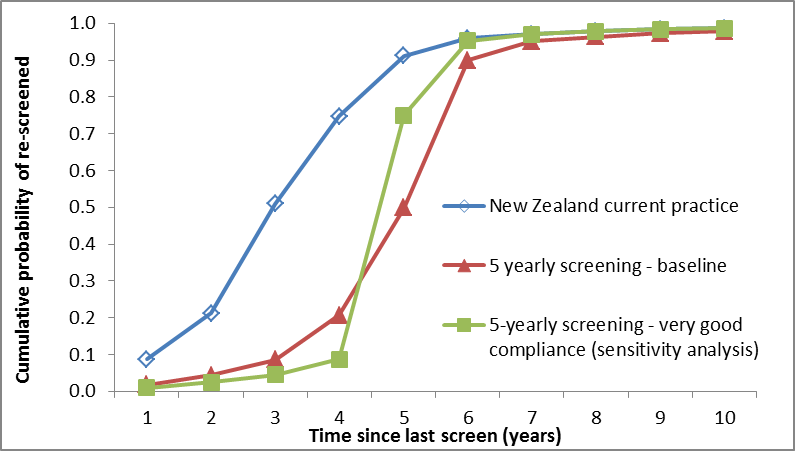


## Screening, diagnosis and treatment cost

We incorporated local cost of cytology test, HPV test (under low-volume usage when cytology is used as primary screening test for women aged 20-69 years), colposcopy with/without histopathology evaluation and treatment for CIN, cancer treatment and terminal care for cancer patient in the model (Table 10). The 2014 screening and diagnosis cost and inflation rate in NZ were based on NCSP opinion. The costs were inflated by an annual rate of 1% to 2017/18 value. HPV test cost was assumed to be $35 in primary HPV screening strategies when the volume of HPV test usage was higher.

Table 10 Modelled aggregated cost for screening, diagnosis and treatment

| **Cost item** | **Cost in 2017** |
| --- | --- |
| **Cost of screening** | |
| Cytology test | $31.10 |
| HPV test (low-volume) | $43.56 |
| HPV test (high-volume) | $35.00 |
| **Cost of diagnosis and treatment for CIN** | |
| Having a colposcopy without histology evaluation | $276.12 |
| Having a colposcopy with histology evaluation and/or treatment for CIN | $567.24 |
| **Cost of cancer treatment** | |
| *Localised cancer* |  |
| 1st year cost | $14,349.86 |
| Annual cost in the subsequent year | $125.71 |
| *Regional cancer* |  |
| 1st year cost | $38,200.35 |
| Annual cost in the subsequent year | $1,186.89 |
| *Distant cancer* |  |
| 1st year cost | $36,188.93 |
| Annual cost in the subsequent year | $3,651.25 |
| **Cost of cancer terminal care** | |
| *Localised cancer* |  |
| For the first year after cancer diagnosed | $21,040.42 |
| For the other years after cancer diagnosed | $11,787.52 |
| *Regional cancer* |  |
| For the first year after cancer diagnosed | $44,942.68 |
| For the other years after cancer diagnosed | $19,629.84 |
| *Distant cancer* |  |
| For the first year after cancer diagnosed | $49,512.75 |
| For the other years after cancer diagnosed | $26,096.70 |

The model also incorporated the costs of inpatient treatment of invasive cervical cancer by extent of disease and time since diagnosis for each of the first five years after diagnosis. The cost was derived by analysing a linked dataset of New Zealand’s National Minimum Dataset and Cancer Register with complete databases of deaths and publicly-funded hospital treatments [33] and calibrated to an average cost of $23,116 per cancer patient reported in 2008/09 in NZ [34]. The cost assumption was inflated from 2008/09 to 2013/14 value according to the consumer price index reported in NZ in that period of time [35,36] and further inflated to 2017/18 value with an annual rate of 1%. We assumed no further costs were incurred for cancer survivor after 10 years since cancer was diagnosed.

The modelled base case cost assumptions are summarised in Table 10. Alternative cost assumptions for cytology test ($25-35) and HPV test under high volume usage ($30-40) were evaluated in sensitivity analysis.

## QALY weights

The cost-effectiveness analysis of this evaluation was focus on life-years saved associated with the primary HPV testing strategy in relation to current practice. Quality adjusted life-years (QALY) saved are evaluated as a secondary outcome. Three set of QALY assumptions vary by different health utilities associated with screening, diagnostic procedures, treatment for precancerous lesions were evaluated. All QALY weight set assumed healthy women without cervical cancer have a health state utility QALY score of 1. Women with cancer detected in the model were assumed to have a stage- and interval- specific mortality rate for a period of 10 years after the cancer was diagnosed. Cancer patients diagnosed with localised, regional and distant cervical cancer were assumed to be associated with a QALY of 0.76, 0.67 and 0.48, respectively for a period of 10 years in the all QALY weight sets. [37][38]. Women who survive 10 years after cancer diagnosis were assumed to become ‘cancer survivors’. It was further assumed that the quality of life of cancer in this group is the same as in the general population (i.e. QALY weight=1); (consistent with some other published studies [39,40]).

### QALY weights set 1

The QALY weights set one were based on the mean estimates from a study that conducted at metropolitan Sydney, New South Wales (NSW), which measured QALY weights via a two-stage standard gamble [41]. This set of weights assigned some disutility to the experience of being screened, even if the test result was negative (based on the results of the study) and small amount disutility to having an abnormal screening outcome (Table 11).

Table 11 Pathway-based QALY weights set one for precancerous cervical lesions and associated investigations

| **Health state** | **Duration (years)** | **Health state preference score** | **Source** |
| --- | --- | --- | --- |
| Cytology normal and HPV negative/no HPV test (‘experience of being screened even if the result is negative’) | 1 | 0.9967 | [41] |
| Cytology normal and HPV positive | 1 | 0.9733 | [41] |
| Cytology abnormal and HPV negative/no HPV test ^a^ | 1 | 0.9735 | [41] |
| Cytology abnormal and HPV positive | 1 | 0.9733 | [41] |
| Colposcopy examination with/without biopsy but do not required treatment for precancerous lesion ^b^ | 1 | 0.9724 | [41] |
| Treatment for precancerous lesion ^c^ | 1 | 0.9704 | [41] |
| Cancer |  |  |  |
| Localised | 10 | 0.76 | [37] |
| Regional | 10 | 0.67 | [37] |
| Distant | 10 | 0.48 | [38] |
| Cancer survivor | 1 | 1 | Assumption |

^a^ Based on the value of LG cytology [41]

^b^ Based on the value of LG cytology with colposcopy normal and HG cytology with CIN1 [41]

^c^ Based on the value of HG cytology with CIN2 or 3 [41]

### QALY weights set 2

QALY weights set two was based on the findings of published studies [37,38], which were not obtained in context of health state preference assessment specifically for primary HPV testing. This set of weights did not assign any disutility to the experience of being screened *per se* (Table 12) but assigned a high for having an abnormal screening outcome compared to QALY weights set one assumption (Table 11).

Table 12 QALY weights set two for precancerous cervical lesions and associated investigations

| **Health state** | **Duration (years)** | **Health state preference score** | **Source** |
| --- | --- | --- | --- |
| False positive | 1 | 0.92 | [42] |
| CIN1 | 1 | 0.89 | [42] |
| CIN2 | 1 | 0.88 | [42] |
| CIN3 | 1 | 0.89 | [42] |
| Cervical Cancer |  |  |  |
| Localised | 10 | 0.76 | [37] |
| Regional | 10 | 0.67 | [37] |
| Distant | 10 | 0.48 | [38,43] |
| Cancer survivor | 1 | 1 | Assumption |

### QALY weights set 3

QALY weights set 3 three (Table 13) was derived based on the findings of Drolet et al. 2012 [44], which compared the QALY in women with normal cytology result with women with abnormal cytology result using EQ-5D, VAS and SF-6D to estimate the lost following an abnormal smear result. This set of weights did not assign any disutility to the experience of being screened *per se* and the disutility assigned for having an abnormal screening outcome was the lowest among the three QALY weights sets assessed in this study.

| **Health state** | **Duration (years)** | **Health state preference score** | **Source** |
| --- | --- | --- | --- |
| CIN1 or low-grade cytology | 1 | 0.996 | [44] |
| CIN2/3 or high-grade cytology | 1 | 0.99 | [44] |
| Cervical Cancer |  |  |  |
| Localised | 10 | 0.76 | [37] |
| Regional | 10 | 0.67 | [37] |
| Distant | 10 | 0.48 | [38] |
| Cancer survivor | 1 | 1 | Assumption |

Table 13 QALY weights set three for precancerous cervical lesions and associated investigations

## Vaccination coverage

The HPV immunisation programmes in NZ, began on 1^st^ of September in 2008, offering free HPV vaccination for girls born in 1990 onwards, with school-based immunisation commencing in 2009 [45]. Girls and young women are eligible for the free HPV vaccine after they turn into 12 years-old and until their 20^th^ birthday [46].

The cumulative dose-2 and dose-3 vaccination uptake in girls and young women born in period 1990 to 1999 as at December 2012 was derived from the coverage data published by Ministry of Health [46] and the program delivery schedule [47] (Figure 42). As limited uptake data were available for cohorts born after 1999, these were assumed to have similar vaccination uptake as the cohort born in 1999. The length of vaccine protection effect of was assumed to be life-long in this evaluation. In the base case analysis, we assumed only girls and young women who have completed 3 doses HPV vaccination will receive the protection effect of the vaccine. A scenario assuming two doses vaccine is as effective as 3 doses vaccine was explored in sensitivity analysis.

Figure 42 Overall coverage rate observed in NZ as at December 2012

# MODEL CALIBRATION AND VALIDATION

## Calibration of natural history model without screening

### HPV prevalence

The natural history model incorporates different rates of disease progression and regression depending on whether the woman is infected with HPV type 16 with or without co-infections of other HPV type (HPV 16), type 18 but not 16 and with or without co-infections of other HPV type (HPV 18) or other non-16/18 oncogenic types (HPV OHR). Due to the absence of local data, the model predicted all oncogenic HPV prevalence and HPV 16 prevalence was calibrated to the data obtained from a population of 805 non-Indigenous, cytologically-normal women attending for routine screening who were recruited to the Women, Human Papillomavirus Prevalence, Indigenous, Non-Indigenous, Urban, Rural Study (WHINURS) [48]. The target prevalence of HPV 18 and HPV OHR for calibration were estimated from the prevalence of all oncogenic HPV observed in WHINURS study and assuming the age-specific HPV type proportion as observed among cytologically-normal women who tested positive by Hybrid-Capture 2 HPV test in a UK trial [49].

Figure 43 shows the comparison of model prediction with the target HPV prevalence data. The model compares well with the target data for most ages. However, it is calibrated to fit to a higher prevalence in the younger age-groups to allow better fitting to other downstream New-Zealand specific targets, including cytology- and histologically-confirmed abnormalities rate among women participate in screening.

Figure 43 (a) Predicted age-specific overall oncogenic HPV prevalence; (b) predicted prevalence of HPV 16, HPV 18 (not 16) and other oncogenic HPV types (not 18 and 16) among sexually active women, compare to the observed data in Australia (WHINURS)

|  |
| --- |
| 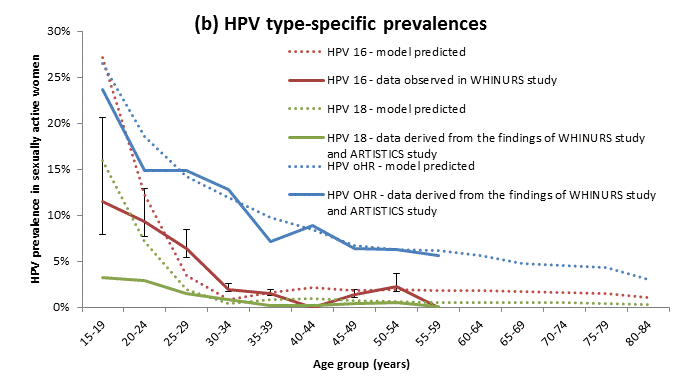 |

Note: HPV prevalence illustrated in the above graphs indicate HPV prevalence before the effect of HPV vaccination takes place. Target prevalence for HPV 18 (not 16) and oncogenic HPV (not 16 or 18) were estimated using the type proportion observed in a UK trials (ARTISITIC)[49].

### Cervical cancer incidence in unscreened population

The average age-specific incidence of cervical cancer in 25 developing countries without significant levels of cervical screening was estimated from data of Cancer incidence in Five Continent published by International Agency of Research on Cancer (IARC) [50] (but it should be noted that a wide range of rates are observed in these countries). The modelled cancer incidence in the absence of screening is broadly consistent with the observed data (Figure 44). Over the age of 50 years, the model predicts a plateau in age-specific cancer incidence; the decrease observed in the data from developing countries is likely to reflect cohort effects in women over 65 years of age (owing to a lesser risk of exposure to HPV infection as younger women). The predicted cumulative lifetime risk of cervical cancer was 3.8% in an unscreened screening among women without hysterectomy.

Figure 44. Predicted age-specific incidence of cervical cancer in unscreened setting, compared to data from 25 developing countries

## Calibration of the screening model

Following the calibration of the natural history model, a complete model of screening, diagnosis and management in NZ was implemented. The full model was of considerable complexity and incorporated data on age-specific screening initiation, compliance with screening, management recommendations in NZ women (informed by an analysis of data obtained from NCSP-R) and the estimates of test characteristics of LBC, HPV test and colposcopy.

The output of the full screening model was compared with:

- The age-specific and age-standardised rate of cervical cancer incidence in NZ in 2008–2012 [51,52,53]
- The age-specific and age-standardised rate of cervical cancer mortality in NZ in 2006-2010 [51,52,54,55,56,57]
- The distribution of cancer stage at diagnosis by age in NZ as observed during the period 1994-2007 [12]
- The distribution of HPV types among new diagnosed cancer cases were calibrated to the findings of a cross-sectional, multicentre hospital-based study conducted in NZ by Skyes et al. [58]
- The age-specific rate of histologically-confirmed high-grade and low-grade as observed in NZ during the period 2010-2011[22]
- The distribution of HPV types among women with a high-grade cytology result and histologically-confirmed high-grade lesion, compared to the observed data in NZ obtained from the Women and HPV study [59]

### Cervical cancer incidence and mortality

The model is calibrated to cervical cancer incidence [51,52,53] and cervical cancer mortality rate [51,52,54,55,56,57] observed in NZ in the period between 2006 and 2012. Figure 45 and Figure 46 shows the model predicted incidence and mortality rates are in close agreement with the observed data.

Figure 45. Predicted age-specific cervical cancer incidence rate per 100,000 women, compared to the observed data in NZ in the period between 2008 and 2012

Figure 46. Predicted age-specific cervical cancer mortality rate per 100,000 women, compared to the observed data in NZ in the period between 2006-2010

### Cervical cancer stage distribution

The model cancer natural history assumption is also calibrated so that the predicted age-specific stage distribution among diagnosed cases are in agreement with the data observed in NZ in the period between 1994 and 2007 [12].

Figure 47 Predicted age-specific cervical cancer stage distribution, compared to data observed in NZ in the period between 1994 and 2007


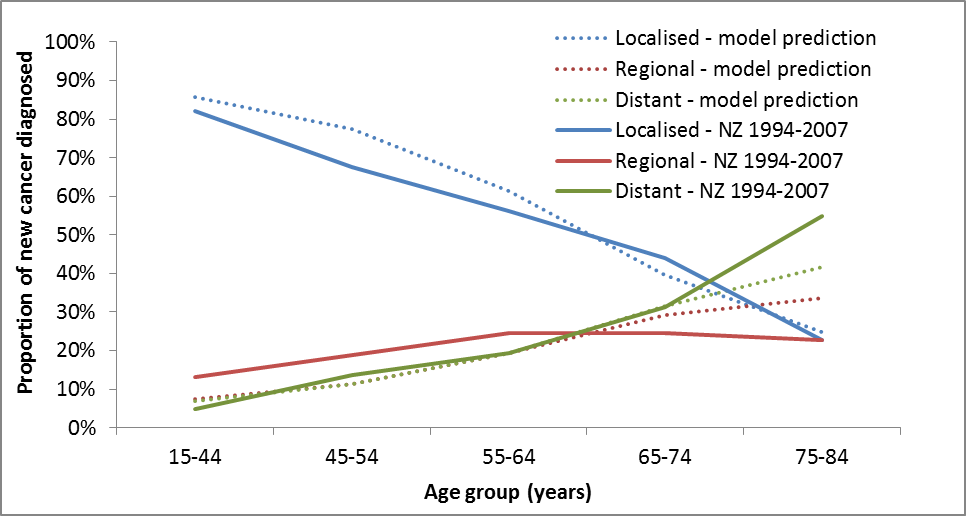


### HPV type distribution in cervical cancer

The modelled HPV type distributions among new diagnosed cancer cases are calibrated to data derived from the findings of a cross-sectional, multicentre hospital-based study conducted in NZ by Sykes et al[58] (Figure 48). The modelled value shows close agreement with the observed data.

Figure 48 Predicted HPV type distribution among diagnosed cancer cases, compared to data observed in NZ


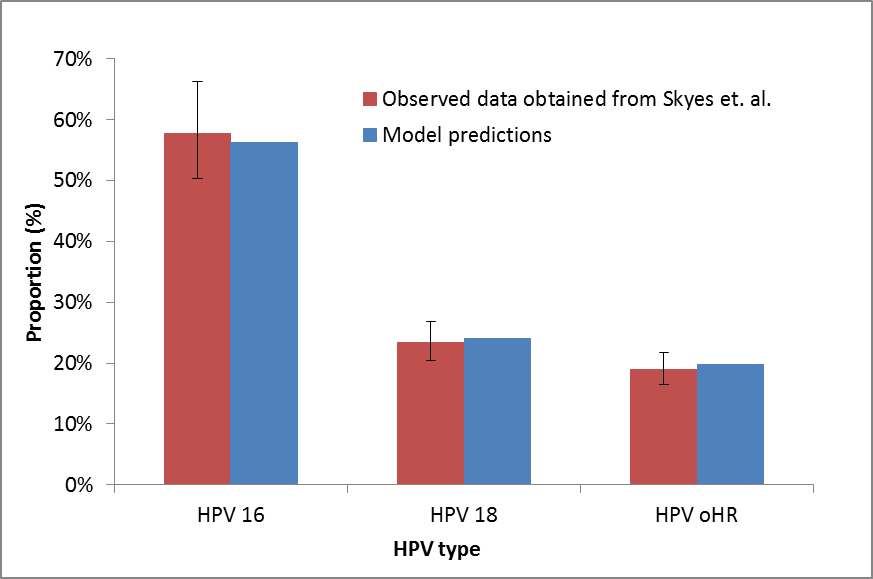


### Histologically-confirmed abnormalities

Model predicted histologically-confirmed abnormality rates are calibrated to data observed in NZ. Figure 49 and Figure 50 compare the predicted histologically-confirmed low-grade rate and high-grade rate per 1,000 women screened with data observed in 2010 and 2011 in NZ [22]. The modelled value shows close agreement with the observed data.

Figure 49 Predicted age-specific rate of histologically-confirmed low-grade lesion per 1,000 women screened, compared to the observed 2010-11 data in NZ

Figure 50 Predicted age-specific rate of histologically-confirmed high-grade lesion per 1,000 women screened, compared to the observed 2010-11 data in NZ

### HPV type distribution among women diagnosed with high-grade lesion

The predicted HPV type distribution among women with a high-grade histology result were also calibrated to the a cross-sectional study which looked the HPV genotype distribution among women participated in National Cervical Screening Programme with a notified high-grade cytology result in NZ in 2009-2011[59]. Figure 51 shows the model prediction is in agreement with the study’s finding among women with histologically confirmed CIN2 or worse.

Figure 51 Predicted HPV type distribution among women with high-grade cytology result and histologically-confirmed high-grade lesion, compared to the observed data in NZ obtained from the Women and HPV study

### Comparing other model’s predictions with data observed in NZ

Table 14 below compares model predictions to other observed data for cervical cancer and screening outcomes. Many of the health outcomes compare well with observed targets from NZ. Predicted annual number of women with high-grade histology and annual number of cytology tests are slightly below the target shown in the table. The number of women with a histology sample collected per year is predicted to be below the target value (12,616 predicted versus 19,230 observed in 2010, or 18,829 observed in 2011 [22]). As many of the other predicted outcomes compare well with observed targets for NZ, there may be other reasons for why the target number of histological evaluations is higher, including histology sample analysed for reasons other than suspicion of cervical abnormalities, some women may have multiple histology evaluations, more women with low-grade cytology outcome underwent colposcopy and histology evaluation compared to what was modelled, double counting for multiple samples per case (some labs will assign a new unique accession for each etc.

Table 14. Model prediction versus actual data for cervical cancer and screening outcome

|  |
| --- |

| **Parameter** | **Model predicted** | **Data observed in NZ** | **Reference** |
| --- | --- | --- | --- |
| Cumulative lifetime risk of cervical cancer, 0-84 years | 0.65% | 0.66% (range: 0.55% - 0.70%) average 2008-2012 | [51,52,53] |
| ASR of cervical cancer incidence per 100,000 women, 0-84 years^*^ | 6.1 | 6.4 (range: 5.4 -7.1) average 2008-2012 |  |
| ASR of cervical cancer incidence per 100,000 women, 20-69 years^*^ | 9.2 | 9.9 (range: 8.3 - 11.1) average 2008-2012 |  |
| Cervical cancer cases, 0-84 years^#^ | 154 | 157 (range: 134 - 175) average 2008-2012 |  |
| ASR of cervical cancer mortality per 100,000 women, 0-84 years^*^ | 1.5 | 1.7 (range: 1.3 - 2.1) average 2006-2010 | [51,52,54,55,56,57] |
| ASR of cervical cancer mortality per 100,000 women, 20-69 years^*^ | 2.1 | 2.2 (range: 1.6 -2.7) average 2006-2010 |  |
| Cervical cancer deaths, 0-84 years^#^ | 43 | 51 (range: 41 - 58) average 2006-2010 |  |
| ASR of histologically-confirmed HG rate per 1,000 women screened, 20-69 years^*^ | 11.8 | 10.9 (2010)  11.9 (2011) | [22] |
| Number of HG histology cases, 20-69 years | 3,816 | 4,188(2010)  4,426(2011) |  |
| ASR of histologically-confirmed LG rate per 1,000 women screened, 20-69 years^*^ | 8.4 | 7.8 (2010)  8.0 (2011) |  |
| Number of LG histology cases, 20-69 years^#^ | 2,768 | 3,048 (2010)  3,092 (2011) |  |
| Number of cytology tests, 0-84 years^#^ | 422,099 | 425,958 (2010)  422,327 (2011) |  |
| Number of cytology tests, 20-69 years^#^ | 390,755 | 410,367 (2010)  406,012 (2011) |  |
| Number of HPV test, 0-84 years^#^ | 24,528 | Not available | Not available |
| Number of HPV test, 20-69 years^#^ | 23,269 | Not available | Not available |
| Number of women underwent colposcopy examination, 0-84 years^#^ | 26,828 | Not available | Not available |
| Number of women underwent colposcopy examination, 20-69 years^#^ | 25,752 | Not available | Not available |
| Number of women with histology samples collected, 20-69 years | 12,233 | 19,230 (2010)  18,829 (2011) | [22] |
| Number of women treated for pre-cancerous lesion,0-84 years^#^ | 4,605 | Not available | Not available |
| Number of women treated for pre-cancerous lesion, 20-69 years^#^ | 4,464 | Not available | Not available |

HG- high-grade; LG-low-grade

**Standardised to WHO population*

*^#^NZ 2012 population was used in calculation of the predicted case number*

## Calibration of cytology test performance assumption

### Test yield

In order to model the local test performance of liquid-based cytology in NZ, the assumptions on the cytology test accuracy were derived by calibrated to the cytological abnormalities rate observed in NZ in 2010-11[22]. Table 1 and Figure 31 show that the model predictions of overall and age-specific cytology tests yield are in close agreement with recent data observed in NZ [22].

### Positive predictive value of high-grade cytology

The cytology test performance is also calibrated to the observed positive prediction value report in 2010-2011[22]. The comparison between the model prediction and data observed in NZ is shows in Table 2.

## Calibration of screening participation assumption

We analysed NCSP register data to determine the proportion of women who re-attend for a smear given their last test result. The model predictions are in close agreement with the 3-year and 5-year screening coverage observed in NZ in 2012 [19] (Figure 39 and Figure 40).

# SUPPLEMENTARY ANALYSIS

QALYs was measured as a secondary outcome in this evaluation using three sets of QALYS weights assumptions:

- QALY weight set 1 assigned some disutility to the experience of being screened, even if the test result was negative (based on the results of the study) and small amount disutility to having an abnormal screening outcome (Table 11).
- QALY weight set 2 did not assign any disutility to the experience of being screened but assigned a high disutility for having an abnormal screening outcome compared to QALY weight set one assumption (Table 12).
- QALY weight set 3 did not assign any disutility to the experience of being screened and the disutility assigned for having an abnormal screening outcome was the lowest among the three QALY weight set assessed in this study (Table 13).

The outcomes of the secondary analysis are show in Table 15 and in Figure 52, Figure 53 and Figure 54.

Table 15 Supplementary cost-effectiveness analysis using QALY as strategies’ effectiveness indicator

| **Strategies** | **Cost** ^a^ | ***Using QALY(set 1) assumption*** | | ***Using QALY(set 2) assumption*** | | ***Using QALY(set 2) assumption*** | |
| --- | --- | --- | --- | --- | --- | --- | --- |
|  |  | **QALYs** ^a^ | **CER relative to CP** | **QALYs** ^a^ | **CER relative to CP** | **QALYs** ^a^ | **CER relative to CP** |
| *Unvaccinated scenario* | | | | | | | |
| **CP** | $533.03 | 34.8368 | - | 34.8371 | - | 34.8697 | - |
| **S1a** | $468.04 | 34.8424 | Cost saving ^*^ | 34.8334 | Less Eff. | 34.8690 | Less Eff. |
| **S1b** | $515.61 | 34.8436 | Cost saving ^*^ | 34.8325 | Less Eff. | 34.8709 | Cost saving |
| **S1c** | $502.88 | 34.8384 | Cost saving ^*^ | 34.8340 | Less Eff. | 34.8688 | Less Eff. |
| **S1d** | $521.61 | 34.8388 | Cost saving ^*^ | 34.8337 | Less Eff. | 34.8696 | Less Eff. |
| **S2a** | $510.54 | 34.8444 | Cost saving ^*^ | 34.8324 | Less Eff. | 34.8716 | Cost saving |
| **S2b** | $542.58 | 34.8443 | $1,279 | 34.8312 | Less Eff. | 34.8721 | $4,032 |
| **S2c** | $506.77 | 34.8390 | Cost saving ^*^ | 34.8342 | Less Eff. | 34.8696 | Less Eff. |
| **S2d** | $524.04 | 34.8389 | Cost saving ^*^ | 34.8337 | Less Eff. | 34.8699 | Cost saving |
| **S3a** | $581.30 | 34.8394 | $18,788 | 34.8278 | Less Eff. | 34.8686 | Less Eff. |
| **S3b** | $627.51 | 34.8405 | $25,573 | 34.8269 | Less Eff. | 34.8704 | $126,463 |
| **S3c** | $581.48 | 34.8366 | Less Eff. | 34.8309 | Less Eff. | 34.8685 | Less Eff. |
| **S3d** | $599.66 | 34.8370 | $513,865 | 34.8306 | Less Eff. | 34.8693 | Less Eff. |
| **S4a** | $627.13 | 34.8415 | $20,258 | 34.8270 | Less Eff. | 34.8712 | $61,416 |
| **S4b** | $657.97 | 34.8413 | $27,788 | 34.8259 | Less Eff. | 34.8717 | $63,322 |
| **S4c** | $588.24 | 34.8374 | $99,390 | 34.8312 | Less Eff. | 34.8695 | Less Eff. |
| **S4d** | $604.91 | 34.8373 | $153,000 | 34.8306 | Less Eff. | 34.8698 | $613,704 |
| *Vaccinated scenario* | | | | | | | |
| **CP** | $429.85 | 34.8457 | - | 34.8511 | - | 34.8760 | - |
| **S1a** | $361.52 | 34.8520 | Cost saving | 34.8485 | Less Eff. | 34.8755 | Less Eff. |
| **S1b** | $402.48 | 34.8525 | Cost saving | 34.8473 | Less Eff. | 34.8767 | Cost saving |
| **S1c** | $396.73 | 34.8477 | Cost saving | 34.8487 | Less Eff. | 34.8753 | Less Eff. |
| **S1d** | $414.82 | 34.8479 | Cost saving | 34.8483 | Less Eff. | 34.8759 | Less Eff. |
| **S2a** | $372.79 | 34.8532 | Cost saving | 34.8484 | Less Eff. | 34.8767 | Cost saving |
| **S2b** | $408.85 | 34.8530 | Cost saving | 34.8471 | Less Eff. | 34.8772 | Cost saving |
| **S2c** | $393.39 | 34.8482 | Cost saving | 34.8490 | Less Eff. | 34.8757 | Less Eff. |
| **S2d** | $412.14 | 34.8481 | Cost saving | 34.8484 | Less Eff. | 34.8760 | Less Eff. |
| **S3a** | $476.28 | 34.8491 | $13,801 | 34.8433 | Less Eff. | 34.8751 | Less Eff. |
| **S3b** | $515.99 | 34.8495 | $22,487 | 34.8422 | Less Eff. | 34.8762 | $533,508 |
| **S3c** | $476.36 | 34.8460 | $186,991 | 34.8456 | Less Eff. | 34.8751 | Less Eff. |
| **S3d** | $493.89 | 34.8461 | $161,885 | 34.8452 | Less Eff. | 34.8756 | Less Eff. |
| **S4a** | $489.26 | 34.8502 | $13,141 | 34.8433 | Less Eff. | 34.8763 | $260,611 |
| **S4b** | $524.05 | 34.8501 | $21,649 | 34.8420 | Less Eff. | 34.8767 | $130,891 |
| **S4c** | $474.35 | 34.8464 | $61,171 | 34.8460 | Less Eff. | 34.8755 | Less Eff. |
| **S4d** | $492.49 | 34.8463 | $98,264 | 34.8454 | Less Eff. | 34.8758 | Less Eff. |

Eff – effective;

* More effective and cost-saving

When assuming QALY weight set, most of the primary HPV screening strategy was predicted to associate with higher QALYs (more effective) than current practice in both unvaccinated and vaccinated scenarios. Strategy S1a (cost saving compared to CP) followed by S2a (ICER: $20,602 per QALY saved in unvaccinated scenario; ICER: $9,769 in vaccinated scenario) were identified as dominating strategy in both scenarios (Figure 52); S2a is identified as the most cost-effective scenario under the indicative willingness-to-pay threshold of $20,000-$50,000 per QALY saved for New Zealand.

Figure 52 Cost-effectiveness plane in (a) unvaccinated scenario and (b) vaccinated scenario, assuming QALY weight set 1

| 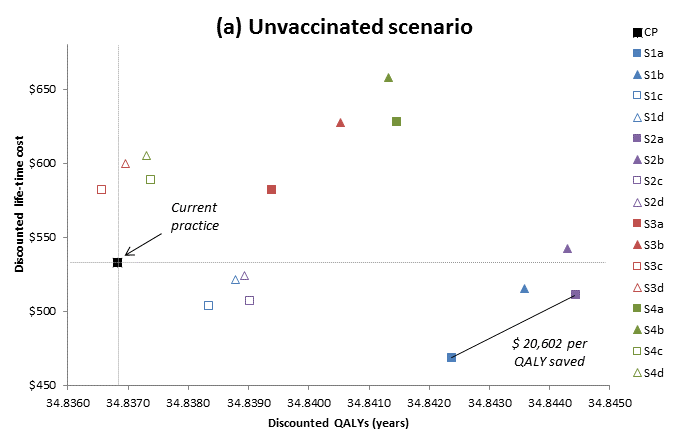 |
| --- |
| 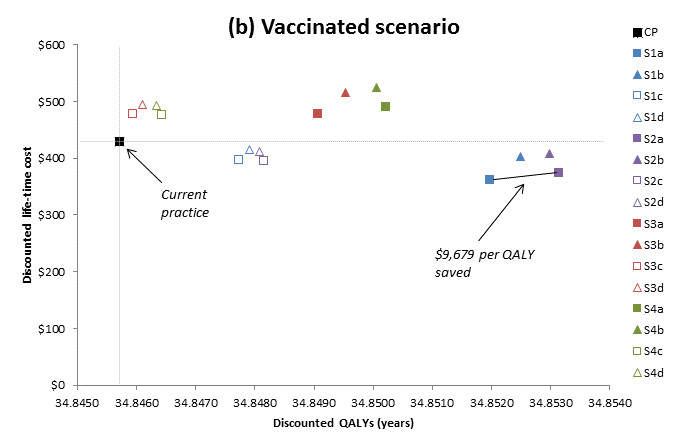 |

When assuming QALY weight set 2 (no disutility to the experience of being screened per se but high disutility for having abnormal screening outcome), all primary HPV screening strategies were predicted to be less effective than current practice in both scenarios (Figure 53); as in past similar evaluations [3,4], this is likely because in QALY Set 2 a high disutility for a test-positive screening outcome was assumed; this was obtained from a small study in university-age women but assumed use of cytology rather than primary HPV screening.

Figure 53 Cost-effectiveness plane of (a) unvaccinated scenario and (b) vaccinated scenario, assuming QALY weight set 2

| 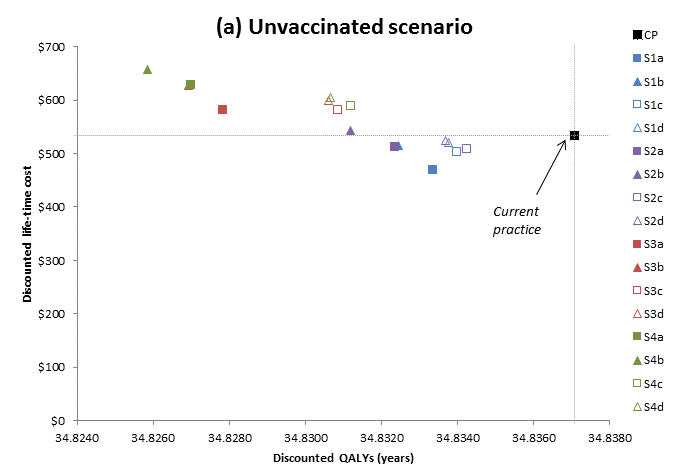 |
| --- |
| 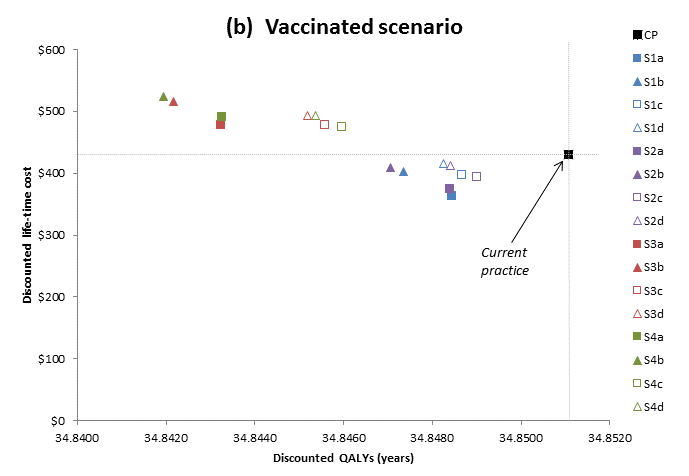 |

Many of the primary HPV screening strategies were predicted to associate with higher QALYs (more effective) than current practice in both scenarios when using QALY weight set 3, which assumed no disutility to the experience of being screened and small amount of disutility from having an abnormal screening outcome. Strategy S2a (cost saving compared to CP) followed by S2b (ICER: $69,221 per QALY saved in unvaccinated scenario; ICER: $70,031 in vaccinated scenario) were identified as dominating strategy in both scenarios (Figure 54); S2a is identified as the most cost-effective scenario under the indicative willingness-to-pay threshold for New Zealand.

Figure 54 Cost-effectiveness plane of (a) unvaccinated scenario and (b) vaccinated scenario, assuming QALY weight set 3

| 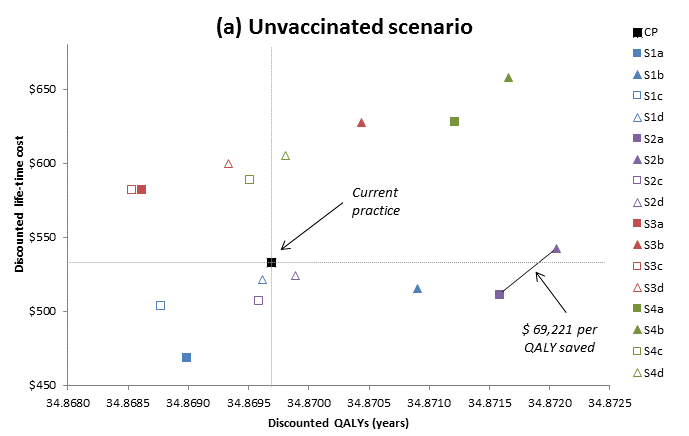 |
| --- |
| 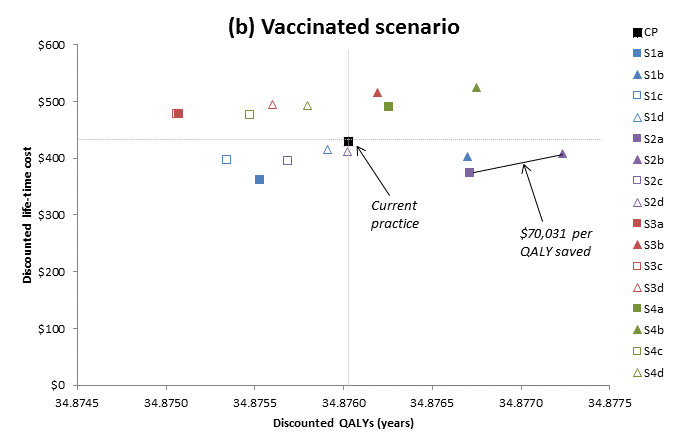 |

The primary HPV screening strategies and current practice were estimated to have a less than 0.002 difference in the estimated life-years. Because of this small difference in the life-years predictions, the QALY findings were sensitive to the QALY weight assumptions used, and hence, varied significantly when different QALY weight sets were assumed in the analysis.

Compared to current practice (3-yearly cytology screening), women undergoing 5-yearly primary HPV screening were estimated to have substantially less screening/follow-up tests (on average ~14 screening/follow-up episodes for current practice vs. 10-11 episodes for primary HPV screening in both unvaccinated and vaccinated scenarios) but a slightly higher number of colposcopy examinations (as well as higher number of abnormal screening outcomes) (1.0 colposcopy examination for current practice vs. 0.9-1.3 colposcopy examinations for primary HPV screening strategies in unvaccinated scenario; 0.7 vs. 0.6-0.9 colposcopy examinations in vaccinated scenario) throughout their lifetime. Therefore, almost all primary HPV screening strategies were predicted to be more effective (associated with more QALYs) than current practice when a small amount of disutility for being screened was incorporated (Figure 52), and all primary HPV screening strategies were predicted to be less effective when a high disutility was assumed for abnormal screening outcome and no disutility for negative screening outcome (QALY weights set 2)(Figure 53).

In response to this issue we have also assessed the impact of a third published set of QALY weighs (QALY set 3 – see below), which assumes no disutility for having a negative screening outcome and small disutility for having an abnormal screening outcomes, about half of the primary HPV screening strategies were predicted to remain more effective than current practice (Figure 54).

Except when QALY weights set 2 was assumed (for which all primary HPV screening strategy were predicted to be less effective than current practice), strategy S2a remained the most cost-effective (and cost-saving) strategy in both the unvaccinated and vaccinated scenarios when QALY weight set 1 and 3 were used.

# SENSITIVITY ANALYSIS

Univariate sensitivity analysis was conducted using S2a and S2c in both scenarios. The parameters and the range included in the sensitivity analysis is summarised in Table 16.

Table 16 Parameter selected for sensitivity analysis

| **Model parameters** | **Baseline assumption** | **Sensitivity analysis range** | |
| --- | --- | --- | --- |
|  |  | **Min** | **Max** |
| Unsatisfactory rate of cytology | 1.17% | 0.60% | 2% |
| Test accuracy of cytology for triaging women with positive HPV result, in 20-69 years ^a,b^ | Sensitivity: 78.7%; Specificity: 74.5% | N/A | *(Best case assumption)* Sensitivity: 84.0%; Specificity: 74.5% |
| Test accuracy of HPV testing for primary screening, in 20-69 years ^b^ | Sensitivity: 96.4%; Specificity: 90.3% | *(Worst case assumption)* Sensitivity: 93.4% ; Specificity: 90.3% | *(Best case assumption)* Sensitivity: 98.6% ;  Specificity: 92.6% |
| Test accuracy of HPV testing for triaging women with ASC-US cytology result, in 20-69 years ^b,c^ | Sensitivity: 90.8%; Specificity: 72.6% | *(Worst case assumption)* Sensitivity: 89.5%; Specificity: 50.6% | *(Best case assumption)* Sensitivity: 94.4%; Specificity: 72.6% |
| Test accuracy of HPV testing for triaging women with LSIL cytology result, in 20-69 years ^b,c^ | Sensitivity: 94.1%; Specificity: 47.4% | *(Worst case assumption)* Sensitivity: 90.5%; Specificity: 24.6% | *(Best case assumption)* Sensitivity: 97.0%; Specificity: 47.4% |
| Test accuracy of HPV testing for follow-up women treated for HG CIN (TOC), in 20-69 years ^b^ | Sensitivity: 92.5%; Specificity: 82.7% | *(Worst case assumption)* Sensitivity: 85.1%; Specificity: 75.3% | *(Best case assumption)* Sensitivity: 96.7%; Specificity: 86.2% |
| Test accuracy of HPV partial genotyping among women test positive for oncogenic HPV infection | Perfect accuracy in detecting the present of HPV 16/18 infections | *(Alternative assumption 1)* 10% of women infected with HPV 16/18 will be misclassified as infected with HPV OHR | *(Alternative assumption 2)* 10% of women tested false positive against all oncogenic type will be misclassified as infected with HPV 16/18 |
| Colposcopy positive rate | No CIN: 50.2%; CIN 1: 76.5%; CIN 2/3: 88.4%; Cancer: 100.0% | No CIN: 45.2%; CIN 1: 68.9%; CIN 2/3: 79.6%; Cancer : 100.0% | No CIN: 73.8%; CIN 1: 79.2%; CIN 2/3: 90.8%; Cancer: 100% |
| Screening initiation for scenario assuming screening starts from 25 years | Rapid screening uptake at age of 25^d^ | Gradual uptake at age of 25 years ^e^ | N/A |
| Routine screening compliance (applicable only to scenarios assumed 5-yearly screening) | 5-yearly reminder-based | 5-yearly call-and-recall | N/A |
| Compliance to follow-up management | Base case assumption | Overall compliance rate decreased by 10% | Overall compliance rate increased by 10% |
| Compliance to colposcopy referral | Base case assumption | Overall compliance rate decreased by 10% | Overall compliance rate increased by 10% |
| Aggressiveness of CIN natural history | Base case assumption (calibrated to multiple targets) | *(5% less aggressive)* A 5% relative decrease in progression rate and increase in regression rate for all CIN transition probabilities | *(5% more aggressive)* A 5% relative increase in progression rate and decrease in regression for all CIN transition probabilities |
| Adjustment for ‘unmasking effect’ for HPV OHR type (application only to scenarios modelled the effect of HPV vaccination) | Base case assumption (calibrated to a ~8% increase in OHR prevalence) | No adjustment for ‘unmasking effect’ | N/A |
| Cytology test cost | $30.19 | $25.00 | $35.00 |
| HPV test cost (under primary HPV screening strategies) | $35.00 | $30.00 | $40.00 |
| Vaccination coverage rate | Coverage rate for female based on 3-dose data | Coverage rate for female based on 2-dose data | N/A |
| Discounted rate | 3.50% | 1% | 5% |

CIN: Cervical intraepithelial neoplasia; HG: high-grade; HPV OHR: oncogenic HPV type other than HPV 16 or 18; TOC: Test-of-cure;
^a^ Cut-off at ASC-US threshold
^b^ For CIN2+detection

^c^ Same test accuracy was modelled for HPV triage testing for women with both ASC-US and LSIL cytology result. Differences in the test sensitivity and specificity were due to the differences in the mix of underlying health states between women with ASC-US and LSIL cytology result.
^d^ Assume women who have had their first screening test at age <=25 years under current practice would all have their first screening test at age 25 years in the scenario assuming screening starts from 25 years
^e^ Assume a gradual screening initiation in 25-29 years. The proportion of women who have had their first screening test by the age of 30 of the scenario was assumed to be the same as the proportion assumed for current practice.

## Strategy S2a, no vaccination

The strategy was predicted to be cost-saving and more effective than current practice under all assumptions except one scenario which assumed the cost of LBC was reduced to $25 per test (Figure 55). The predicted cost associated with the strategy was found to be sensitive to the assumptions made for the cost of cytology test, the screening uptake patterns, and characteristic of HPV test for primary screening or for triage test on women with low-grade cytology result, the cost of HPV test, the accuracy of HPV partial genotyping and the aggressiveness of natural history. The predicted life-years associated with the strategy was found to be sensitive to the assumptions made for discount rate, the screening uptake patterns, the aggressiveness of natural history, the accuracy of HPV partial genotyping, the compliance with recommendations to return for follow-up screening tests and the characteristic of HPV test for primary screening.

Figure 55 Relative lifetime cost (shown as blue bars on the left) and life-years (shown as red bars on the right) of strategy S2a compared to current practice for each of the items explored in one-way sensitivity analysis – unvaccinated scenario (see footnote 1)

The modelled base case strategy S2a was predicted to be associated with 4.22% reduction in lifetime cost and 0.0041% increase in life-years compared to current practice under no vaccination scenario.

Compared to baseline assumption, the strategy was predicted to be associated with a higher amount of cost saving in relation to current practice when assuming both best case and worst case HPV test characteristics (worst case assumption: -4.3%; best case assumption: -10.8%) the strategy was predicted to be associated with a lower amount of life-years saved in relation to current practice when assuming the worst case test characteristics for HPV test for primary screening and was predicted to be associated with a higher amount of life-years saved in relation to current practice when assuming the best case test characteristics for HPV test for primary screening.

Compared to baseline assumption, the strategy was predicted to be associated with higher amount of cost saving (worst case assumption: -6.82%; best case assumption:-4.22%) and modest increase in life-years saved (worst case assumption: 0.0042%; best case assumption: 0.0041%) in relation to current practice when assuming the both the worst case and best case test characteristics for HPV test for triage women with low-grade cytology outcome.

When compared to baseline assumption, the strategy was predicted to be associated with a higher amount of cost saving in relation to current practice when either 1% or 5% discount rate (1% discount rate: -4.66%; 5% discount rate: -4.62%); the strategy was predicted to be associated with a lower amount of life-years saved in relation to current practice when assuming the 5% discount rate and higher amount when assuming 1% discount rate.

## Strategy S2c, no vaccination

The strategy was predicted to be cost-saving and more effective than current practice under all assumptions included for 1-way sensitivity analysis (Figure 56). The predicted cost associated with the strategy was found to be sensitive to the assumptions made for the cost of cytology test and HPV test, the characteristic of HPV test for primary screening or for triage test on women with low-grade cytology result, the discount rate, the aggressiveness of natural history and the accuracy of HPV partial genotyping. The predicted life-years associated with the strategy was found to be sensitive to the assumptions made for the discount rate, the compliance with recommendations to return for follow-up screening tests, the aggressiveness of natural history, the accuracy of HPV partial genotyping and the characteristic of HPV test for primary screening or for triage test on women with low-grade cytology result.

Figure 56 Relative lifetime cost (shown as blue bars on the left) and life-years (shown as red bars on the right) of strategy S2c compared to current practice for each of the items explored in one-way sensitivity analysis – unvaccinated scenario (see footnote 1)

The modelled base case strategy S2c was predicted to be associated with 4.93% reduction in lifetime cost and 0.00052% increase in life-years compared to current practice under no vaccination scenario

When compared to baseline assumption, the strategy was predicted to be associated with a higher amount of cost saving and life-years saved in relation to current practice when assuming the best case HPV test characteristics; the strategy was predicted to be associated with a lower amount of cost saving and life-years saved in relation to current practice when assuming the worst case HPV test characteristics.

When compared to baseline assumption, the strategy was predicted to be associated with higher amount of cost saving (worst case assumption: -7.39%; best case assumption: -4.93%) and life-years saved (worst case assumption: 0.00061%; best case assumption: 0.00045%) in relation to current practice when assuming the worst case test accuracy of HPV testing for women with low-grade cytology.

When compared to baseline assumption, the strategy was predicted to be associated with a higher amount of cost saving and a life-years saved in relation to current practice in the when assuming 1% discount rate; the strategy was predicted to be associated with a lower amount of cost saving and life-years saved in relation to current practice when assuming 5% discount rate.

When compared to baseline assumption, the strategy was predicted to be associated with higher amount of cost saving and life-years saved in relation to current practice when assuming higher compliance to the 5-yearly screening interval.

When compared to baseline assumption, the strategy was predicted to be associated with a lower amount of cost saving but a higher amount of life-years saved in relation to current practice in the when assuming best case HPV test characteristics; the strategy was predicted to be associated with a lower amount of cost saving and life-years saved in relation to current practice when assuming worst case HPV test characteristics.

When compared to baseline assumption, the strategy was predicted to be associated with a higher amount of cost saving but a lower amount of life-years saved in relation to current practice in the when assuming a higher positive rate for colposcopy; the strategy was predicted to be associated with a higher amount of cost saving and life-years saved in relation to current practice when assuming a lower positive rate for colposcopy.

When compared to baseline assumption, the strategy was predicted to be associated with a modestly higher amount of cost saving (-4.94%) and higher amount of life-years saved in relation to current practice in the when assuming a best case test accuracy of LBC test for triage women with positive HPV test outcome

##

## Strategy S2a, vaccination

The strategy was predicted to be cost-saving and more effective than current practice under all assumptions included for 1-way sensitivity analysis (Figure 57). The predicted lifetime cost associated with the strategy was found to be sensitive to the assumptions made for the discount rate, cost of cytology test and HPV test, the characteristic of HPV test for primary screening or for triage test on women with low-grade cytology result, the screening uptake patterns, the aggressiveness of natural history, and the accuracy of HPV partial genotyping. The predicted life-years associated with the strategy was found to be sensitive to the assumptions made for the discount rate, the aggressiveness of natural history, the screening uptake patterns, the accuracy of HPV partial genotyping, and the characteristic of HPV test for primary screening.

Figure 57 Relative lifetime cost (shown as blue bars on the left) and life-years (shown as red bars on the right) of strategy S2a compared to current practice for each of the items explored in one-way sensitivity analysis – vaccinated scenario (see footnote 1 and 2)

The modelled base case strategy S2a was predicted to be associated with 13.3% reduction in lifetime cost and 0.0018% increase in life-years compared to current practice under vaccination scenario

The strategy is predicted to be associated with a 29.01% reduction in life-time cost and a 0.023% increase in life-years in relation to current practice when assuming 1% discount rate (when compared to baseline assumption, it is estimated to be associated with lower amount of cost saving but higher amount of life-years saved in relation to current practice); the strategy is predicted to be associated with a 31.15% reduction in life-time cost and a 0.0094% increase in life-years in relation to current practice when assuming 5% discount rate (when compared to baseline assumption, it is estimated to be associated with higher amount of cost saving and life-years saved in relation to current practice).

Compared to baseline assumption, the strategy was predicted to be associated with a higher amount of cost saving in relation to current practice when assuming both best case and worst case HPV test characteristics (worst case assumption: -13.3%; best case assumption: -19.2%) the strategy was predicted to be associated with a lower amount of life-years saved in relation to current practice when assuming the worst case test characteristics for HPV test for primary screening and was predicted to be associated with a higher amount of life-years saved in relation to current practice when assuming the best case test characteristics for HPV test for primary screening.

When compared to baseline assumption, the strategy was predicted to be associated with higher amount of cost saving (worst case assumption: -16.0%; best case assumption: -13.3%) and life-years saved (worst case assumption: 0.0019%; best case assumption: 0.0018%) in relation to current practice when assuming the worst case test accuracy of HPV testing for women with low-grade cytology.

When compared to baseline assumption, the strategy was predicted to be associated with a higher amount of cost saving in relation to current practice when either assuming a higher or a lower compliance rate; the strategy was predicted to be associated with a higher amount of life-years saved in relation to current practice when assuming a decreased in compliance rate and was associated with a lower amount of life-years saved when assuming an increased in compliance rate.

## Strategy S2c, vaccination

The strategy was predicted to be cost-saving under all assumptions included for 1-way sensitivity analysis (Figure 58). The strategy was predicted to be more effective than current practice in most cases except when assumed the worst case test accuracy of HPV test for primary screening, imperfect accuracy of partial genotyping (10% of women infected with HPV 16/18 were misclassified as OHR type) and no adjustment for the ‘unmasking effect’. The predicted cost associated with the strategy was found to be sensitive to the assumptions made for the discount rate, the cost of cytology test and HPV test, the characteristic of HPV test for primary screening or for triage test on women with low-grade cytology result, the aggressiveness of natural history, and the accuracy of HPV partial genotyping. The predicted life-years associated with the strategy was found to be sensitive to the assumptions made for the discount rate, the accuracy of HPV partial genotyping, the vaccine coverage, the adjustment for ‘unmasking effect’, and the characteristic of HPV test for primary screening or for triage test on women with low-grade cytology result.

Figure 58 Relative lifetime cost (shown as blue bars on the left) and life-years (shown as red bars on the right) of strategy S2c compared to current practice for each of the items explored in one-way sensitivity analysis – vaccinated scenario (see footnote 1 and 2)

The modelled base case strategy S2c was predicted to be associated with 8.483% reduction in lifetime cost and 0.000034% increase in life-years compared to current practice under vaccination scenario

The strategy is predicted to be associated with a 27.13% reduction in life-time cost and a 0.0198% increase in life-years in relation to current practice when assuming a 1% discount rate; the strategy is predicted to be associated with a 25.77% reduction in life-time cost and a 0.0082% increase in life-years in relation to current practice when assuming a 5% discount rate. When compared to baseline assumption, the strategy is estimated to be associated with higher amount of cost saving and life-years saved in relation to current practice when either assuming a 1% or 5% discount rate.

When compared to baseline assumption, the strategy was predicted to be associated with a higher amount of cost saving and life-years saved in relation to current practice when assuming the best case HPV test characteristics; the strategy was predicted to be associated with a lower amount of cost saving and life-years saved in relation to current practice when assuming the worst case HPV test characteristics.

When compared to baseline assumption, the strategy was predicted to be associated with a higher amount of cost saving (worst case assumption: -11.3%; best case assumption: - 8.5%) and life-years saved (worst case assumption: 0.000092%; best case assumption: -0.000007%) in relation to current practice when assuming the worst case test accuracy of HPV testing for women with low-grade cytology.

When compared to baseline assumption, the strategy was predicted to be associated with a higher amount of cost saving and life-years saved in relation to current practice when assuming higher compliance to the 5-yearly screening interval.

When compared to baseline assumption, the strategy was predicted to be associated with a higher amount of cost saving in relation to current practice when either assuming a higher (-8.7%) or a lower compliance rate (-8.5%); the strategy was predicted to be associated with a higher amount of life-years saved in relation to current practice when assuming a decreased in compliance rate and associate with a lower amount of life-years saved when assuming an increased in compliance rate.

When compared to baseline assumption, the strategy was predicted to be associated with a lower amount of cost saving in relation to current practice when either assuming the best case or the worst case HPV test characteristics; the strategy was predicted to be associated with a lower amount of life-years saved when assuming the best case test characteristics and higher amount of life-years saved when assuming the worst case test characteristics.

When compared to baseline assumption, the strategy was predicted to be associated with a modestly higher amount of cost saving (-8.503%) and higher amount of life-years saved in relation to current practice when assuming a best case test accuracy of LBC test for triage women with positive HPV test outcome

When compared to baseline assumption, the strategy was predicted to be associated with a modestly lower amount of cost-saving in relation to current practice when either assuming a decreased (-8.481%) or an increased (-8.482%) in the compliance rate; the strategy was predicted to be associated with a lower amount of life-years saved when assuming the compliance rate decreased by 10% and a higher amount of life-years saved when assuming the compliance rate increased by 10%

**References**

1. Canfell K, Barnabas R, Patnick J, Beral V (2004) The predicted effect of changes in cervical screening practice in the UK: results from a modelling study. Br J Cancer 91: 530-536.

2. Creighton P, Lew JB, Clements M, Smith M, Howard K, Dyer S, et al. (2010) Cervical cancer screening in Australia: modelled evaluation of the impact of changing the recommended interval from two to three years. BMC Public Health 10: 734.

3. Kitchener HC, Canfell K, Gilham C, Sargent A, Roberts C, Desai M, et al. (2014) The clinical effectiveness and cost-effectiveness of primary human papillomavirus cervical screening in England: extended follow-up of the ARTISTIC randomised trial cohort through three screening rounds. Health Technol Assess 18: 1-196.

4. Lew JB and Simms K, Smith MA, Kang YK, Xu XM, Caruana M, et al. (2014) National Cervical Screening Program Renewal: Effectiveness modelling and economic evaluation in the Australian setting (Assessment Report). MSAC application number 1276. Canberra: Department of Health

5. Medical Services Advisory Committee (2009) Human Papillomavirus Triage Test For Women With Possible or Definite Low-Grade Squamous Intraepithelial Lesions. MSAC reference 39, Assessment report. Canberra: Australian Government Department of Health.

6. Lew J-B, Howard K, Gertig D, Smith M, Clements M, Nickson C, et al. (2012) Expenditure and resource utilisation for cervical screening in Australia. BMC Health Services Research 12: 446.

7. Canfell K, Shi JF, Lew JB, Walker R, Zhao FH, Simonella L, et al. (2011) Prevention of cervical cancer in rural China: Evaluation of HPV vaccination and primary HPV screening strategies. Vaccine 29: 2487-2494.

8. Shi JF, Canfell K, Lew JB, Zhao FH, Legood R, Ning Y, et al. (2011) Evaluation of primary HPV-DNA testing in relation to visual inspection methods for cervical cancer screening in rural China: an epidemiologic and cost-effectiveness modelling study. BMC Cancer 11: 239.

9. Creighton P, Lew J, Clements M, Smith M, Howard K, Dyer S, et al. (2010) Cervical cancer screening in Australia: modelled evaluation of the impact of changing the recommended interval from two to three years. BMC Public Health 10: 734.

10. National Screening Unit (2008) Guidelines for Cervical Screening in New Zealand: Incorporating the management of women with abnormal cervical smears. Wellington: National Screening Unit, Ministry of Health.

11. Smith M, Canfell K, Simonella L, Nickson C, Lew JB, Creighton P, et al. (2009) Economic Evaluation of the Impact of HPV Vaccination on Cervical Screening in New Zealand (report to New Zealand Ministry of Health).Unpublished.

12. New Zealand Health Information Service (2006) Cancer patient survival: Covering the period 1994-2003. Wellington, New Zealand: Ministry of Health.

13. Paul S, Tobias M, Wright C (2005) Setting outcome targets for the National Cervical Screening Programme: A report for the National Screening Unit. Wellington, New Zealand: National Cervical Screening Programme, Ministry of Health.

14. Statistics New Zealand (2006) National population estimates: September 2006 quarter. Wellington, New Zealand: Statistics New Zealand.

15. Smith M, Walker R, Canfell K (2012) National Cervical Screening Programme Monitoring Report Number 34. Wellington.

16. Smith M, Walker R, Canfell K (2012) National Cervical Screening Programme Monitoring Report Number 33. Wellington.

17. Smith M, Walker R, Canfell K (2013) National Cervical Screening Programme Monitoring Report Number 35. Wellington.

18. Smith M, Walker R, Canfell K (2013) National Cervical Screening Programme Monitoring Report Number 36. Wellington.

19. Smith M, Walker R, Canfell K (2013) National Cervical Screening Programme Monitoring Report Number 37. Wellington, NZ: National Screening Unit.

20. Smith M, Walker R, Canfell K (2014) National Cervical Screening Programme Monitoring Report Number 38. Wellington, NZ: National Screening Unit.

21. Smith M, Walker R, Canfell K (2014) National Cervical Screening Programme Monitoring Report Number 39. Wellington: National Screening Unit.

22. Smith M, Walker R, Canfell K (2013) National Cervical Screening Programme Annual Report (2010-2011). Wellington, NZ: National Screening Unit.

23. Smith M, Walker R, Canfell K (2014) National Cervical Screening Programme Monitoring Report Number 40. Wellington: National Screening Unit.

24. Arbyn M, Ronco G, Anttila A, Meijer CJ, Poljak M, Ogilvie G, et al. (2012) Evidence regarding human papillomavirus testing in secondary prevention of cervical cancer. Vaccine 30 Suppl 5: F88-99.

25. Arbyn M, Roelens J, Simoens C, Buntinx F, Paraskevaidis E, Martin-Hirsch P, et al. (2013) Human papillomavirus testing versus repeat cytology for triage of minor cytological cervical lesions. Cochrane Database of Systematic Reviews.

26. Castle PE, Fetterman B, Thomas Cox J, Shaber R, Poitras N, Lorey T, et al. (2010) The age-specific relationships of abnormal cytology and human papillomavirus DNA results to the risk of cervical precancer and cancer. Obstet Gynecol 116: 76-84.

27. Ronco G, Cuzick J, Segnan N, Brezzi S, Carozzi F, Folicaldi S, et al. (2007) HPV triage for low grade (L-SIL) cytology is appropriate for women over 35 in mass cervical cancer screening using liquid based cytology. Eur J Cancer 43: 476-480.

28. Sherman ME, Schiffman M, Cox JT (2002) Effects of age and human papilloma viral load on colposcopy triage: data from the randomized Atypical Squamous Cells of Undetermined Significance/Low-Grade Squamous Intraepithelial Lesion Triage Study (ALTS). J Natl Cancer Inst 94: 102-107.

29. Stoler MH, Wright TC, Jr., Sharma A, Zhang G, Apple R, Wright TL, et al. (2012) The interplay of age stratification and HPV testing on the predictive value of ASC-US cytology. Results from the ATHENA HPV study. Am J Clin Pathol 137: 295-303.

30. Medical Services Advisory Committee (2009) Automation Assisted and Liquid Based Cytology for Cervical Cancer Screening. MSAC reference 1122, Assessment report. Canberra: Australian Government Department of Health.

31. Moss S, Kelly R, Legood R, Sadique Z, Canfell K, Lew JB, et al. (2011) Evaluation of Sentinel Sites for HPV Triage and Test of Cure: Report to NHS Cancer Screening Programmes.

32. Smith M, Walker R, Simms K, Canfell K (2014) National Cervical Screening Programme Annual Report 2012. Wellington, NZ.

33. Canfell K, Clements M, Harris J (2007) Cost-effectiveness of proposed changes to the national cervical screening program. Wellington, New Zealnad: National Screening Unit, New Zealand Ministry of Health.

34. Ministry of Health (2011) The Price of Cancer: The public price of registered cancer in New Zealand. Wellington: Ministry of Health.

35. Statistics New Zealand Tatauranga Aotearoa (2014) Consumers Price Index: June 2014 quarter. Wellington: Statistics New Zealand Tatauranga Aotearoa.

36. Statistics New Zealand Tatauranga Aotearoa (2008) Consumers Price Index: Jan 2008 quarter. Wellington: Statistics New Zealand Tatauranga Aotearoa.

37. Elbasha EH, Dasbach EJ, Insinga RP (2007) Model for assessing human papillomavirus vaccination strategies. Emerging Infectious Diseases 13: 28-41.

38. Goldie SJ, Kohli M, Grima D, Weinstein MC, Wright TC, Bosch FX, et al. (2004) Projected clinical benefits and cost-effectiveness of a human papillomavirus 16/18 vaccine. JNatlCancer Inst 96: 604-615.

39. Kitchener HC, Blanks R, Cubie H, Desai M, Dunn G, Legood R, et al. (2011) MAVARIC - a comparison of automation-assisted and manual cervical screening: a randomised controlled trial. Health Technol Assess 15: iii-xi, 1.

40. de Kok IM, van Rosmalen J, Dillner J, Arbyn M, Sasieni P, Iftner T, et al. (2012) Primary screening for human papillomavirus compared with cytology screening for cervical cancer in European settings: cost effectiveness analysis based on a Dutch microsimulation model. BMJ 344: e670.

41. Simonella L, Howard K, Canfell K (2014) A survey of population-based utility scores for cervical cancer prevention. BMC Res Notes 7: 899.

42. Insinga RP, Glass AG, Myers ER, Rush BB (2007) Abnormal outcomes following cervical cancer screening: Event duration and health utility loss. Medical Decision Making 27: 414-422.

43. Goldie SJ, Kim JJ, Wright TC (2004) Cost-effectiveness of human papillomavirus DNA testing for cervical cancer screening in women aged 30 years or more. ObstetGynecol 103: 619-631.

44. Drolet M, Brisson M, Maunsell E, Franco EL, Coutlee F, Ferenczy A, et al. (2012) The psychosocial impact of an abnormal cervical smear result. Psychooncology 21: 1071-1081.

45. Ministry of Health (2014) History of the HPV immunisation programme. [updated 09 April 2014] Available at: http://www.health.govt.nz/our-work/preventative-health-wellness/immunisation/hpv-immunisation-programme/history-hpv-immunisation-programme. Accessed 25 September 2014.

46. Ministry of Health (2014) HPV immunisation programme. [updated 27 June 2014] Available at: http://www.health.govt.nz/our-work/preventative-health-wellness/immunisation/hpv-immunisation-programme. Accessed 17 February 2015.

47. Ministry of Health (2008) The HPV Immunisation Programme: National Implementation Startegic Oveview. Wellington: Ministry of Health.

48. Garland SM, Brotherton JM, Condon JR, McIntyre PB, Stevens MP, Smith DW, et al. (2011) Human papillomavirus prevalence among indigenous and non-indigenous Australian women prior to a national HPV vaccination program. BMC Med 9: 104.

49. Kitchener HC, Almonte M, Wheeler P, Desai M, Gilham C, Bailey A, et al. (2006) HPV testing in routine cervical screening: cross sectional data from the ARTISTIC trial. Br J Cancer 95: 56-61.

50. Parkin DM, Whelen SL, Ferlay J, Teppo L, Thomas DB (2002) Cancer incidence in five continents Vol. VIII. Lyon, France: IARC Scientific Publications.

51. Ministry of Health (2011) Cancer: New registrations and deaths 2008. Wellington, New Zealand: Ministry of Health.

52. Ministry of Health (2012) Cancer: New registrations and deaths 2009. Wellington: Ministry of Health.

53. Ministry of Health (2014) Cancer: New registrations and deaths 2011. Wellington: Ministry of Health.

54. Ministry of Health (2010) Cancer: New registrations and deaths 2007. Wellington: Ministry of Health.

55. Ministry of Health (2010) Cancer: New registrations and deaths 2006. Wellington: Ministry of Health.

56. Ministry of Health (2009) Cancer: New Registrations and Deaths 2005 Revised edition. Wellington, New Zealand: Ministry of Health.

57. Ministry of Health (2013) Cancer: New Registrations and deaths 2010. Wellington: Ministry of Health.

58. Sykes P, Gopala K, Tan AL, Kenwright D, Petrich S, Molijn A, et al. (2014) Type distribution of human papillomavirus among adult women diagnosed with invasive cervical cancer (stage 1b or higher) in New Zealand. BMC Infect Dis 14: 374.

59. Simonella L, Lewis H, Smith M, Neal H, Bromhead C, Canfell K (2013) The prevalence of type-specific oncogenic human papillomavirus infection in high grade cervical disease in New Zealand. BMC Infect Dis 13.
